# Research progress on the natural products in the intervention of myocardial infarction

**DOI:** 10.3389/fphar.2024.1445349

**Published:** 2024-08-22

**Authors:** Qiuting Guo, Jinhui Wang, Caixia Ni, Jiaojiao Pan, Junbo Zou, Yajun Shi, Jing Sun, Xiaofei Zhang, Deng Wang, Fei Luan

**Affiliations:** ^1^ College of Pharmacy, Xianyang Polytechnic Institute, Xianyang, China; ^2^ Shaanxi Key Laboratory of Chinese Medicine Fundamentals and New Drugs Research, School of Pharmacy, Shaanxi University of Chinese Medicine, Xi’an, Shaanxi, China; ^3^ Key Laboratory of Medicinal and Edible Plants Resources Development of Sichuan Education Department, Sichuan Industrial Institute of Antibiotics, School of Pharmacy, Chengdu University, Chengdu, Sichuan, China; ^4^ Department of Pharmacy, Xi’an No. 3 Hospital, The Affiliated Hospital of Northwest University, Xi’an, Shaanxi, China

**Keywords:** myocardial infarction, molecular mechanisms, natural products, metabolites, medicinal plants

## Abstract

Coronary heart disease is a prevalent cardiovascular ailment globally, with myocardial infarction (MI) being one of its most severe manifestations. The morbidity and mortality of MI are escalating, showing an increasing trend among younger, highly educated individuals, thereby posing a serious threat to public health. Currently, thrombolysis, percutaneous coronary intervention, and coronary artery bypass grafting are the primary clinical treatments for MI. Although these methods significantly reduce patient mortality, complications often result in poor prognoses. Due to limitations in chemical synthetic drug research, the focus has shifted towards developing herbs based on natural substances. Natural medicines represent a novel approach for safer and more effective MI management and treatment. They can control multiple pathogenic variables by targeting various pathways and systems. This paper investigates the molecular mechanisms of MI and evaluates the application of natural products and medicinal plants in MI treatment over the past 5 years, demonstrating their specific good therapeutic potential and superior tolerance. These natural therapies have been shown to mitigate myocardial cell damage caused by MI through mechanisms such as oxidative stress, inflammation, apoptosis, angiogenesis, myocardial fibrosis, autophagy, endoplasmic reticulum stress, mitophagy, and pyroptosis. This review offers the latest insights into the application of natural products and medicinal plants in MI treatment, elucidating their mechanisms of action and serving as an important reference for MI prevention.

## Graphical Abstract

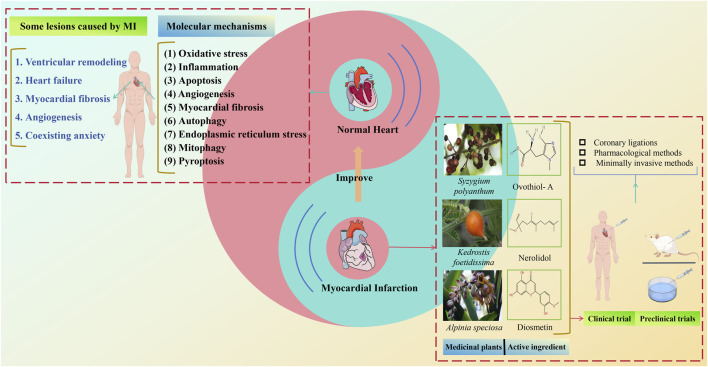

## 1 Introduction

Cardiovascular disease (CVD) remains the leading cause of mortality globally, with approximately seven million deaths in 2020, representing 12.8% of all deaths (Virani et al., 2020; Teo and Rafiq, 2021). Myocardial infarction (MI), or myocardial ischemic injury, is a critical manifestation of CVD, resulting from restricted blood and oxygen supply to the heart (Redline et al., 2023). The presentation of symptoms associated complications, and recurrence risks significantly impact patients’ quality of life and increase mortality rates, particularly sudden death, which is a common outcome of MI (Liu B. et al., 2021a; Liu L. et al., 2021b). MI initiates a progressive decline in cardiomyocyte function, leading to disorganized ventricular wall contractions, reduced pumping efficiency, and adverse cardiac remodeling. This cascade deteriorates heart function, potentially advancing to severe heart failure (HF) or death (Shah et al., 2019). The primary cause of MI is the imbalance between coronary blood flow and the heart muscle’s oxygen demand, prompting cellular changes in MI. This imbalance also generates free radicals and triggers lipid peroxidation, potentially causing irreversible damage to cardiac muscle cell membranes (Saravanan et al., 2013).

Currently, chemical therapies have evolved from basic diuretics and vasodilators to combinations of diuretics, angiotensin-converting enzyme inhibitors, and blockers in clinical practice for MI management. While these drugs are essential in alleviating symptoms, improving hemodynamic parameters, and enhancing myocardial oxygen consumption, they do not address the root causes of MI or its complications. The rising incidence of MI, the lack of effective pharmacological treatments for HF, and the emergence of various complications due to the side effects of chemical drugs present significant challenges. This burden can lead to the development of inhibitory symptoms in patients.

The field of plants offers abundant resources for anti-MI herbs, with traditional medicine globally having a long history and wide application in this area. Notably, natural product therapies are widely recognized as complementary or alternative treatments for MI. Botanical metabolites in these therapies are generally gentler and safer compared to chemically synthesized anti-MI metabolites, attracting significant attention. Millennia of clinical practice have resulted in extensive valuable experience and numerous effective classical natural medicines, especially in traditional Chinese medicine (TCM). Natural products like Quercetin (Patel et al., 2018), Diosmetin (Ahmad et al., 2023), and Celastrol (Fan et al., 2023), have shown significant protective effects on cardiomyocytes. Additionally, natural medicine therapy is popular in traditional medical approaches in countries such as India, South Korea, Australia, Japan, and Mexico, as well as across various African and South American nations.

In the past 5 years, numerous metabolites with potent anti-MI properties have been discovered in natural products. Compounds such as Nerolidol (Gonçalves et al., 2022), Alpha-terpineol (Paulino et al., 2022), and *Syzygium polyanthum* (Hasan et al., 2020), among other medicinal plants, have been extensively documented for their beneficial impacts on MI and its associated complications. However, comprehensive reviews on natural herbs and their combinations for managing MI are scarce. Recent years have seen increased investigations into the use of herbs and plant-based substances for treating MI and its complications. Despite this, a clear gap exists in the research and application of natural products and their active metabolites, which may hinder their optimal use. This article aims to present a comprehensive review of natural products and medicinal plants related to the prevention and treatment of MI over the past 5 years. It will introduce the specific mechanisms of action, and clinical and toxicity studies of various natural products and medicinal plants, based on the pathogenesis and treatment mechanisms of MI. This review seeks to provide a knowledge base for further studies on natural products and medicinal plants in MI treatment and offer a new perspective for enhancing research and discovering new herbs for MI treatment.

## 2 Methods

For this review, we conducted searches on databases including PubMed, Web of Science, China National Knowledge Infrastructure, and ScienceDirect. Keywords such as “medicinal plants,” “natural products,” “molecular mechanisms” and “myocardial infarction” were employed in the search process. The inclusion criteria encompassed global literature specifically addressing natural products and medicinal plants in relation to MI treatment over the past 5 years until January 2024. Each chosen article underwent meticulous scrutiny to extract details on active metabolites, biological effects of medicinal plants, study design, experimental models, dosages, primary outcomes, and mechanisms of action. Studies lacking adequate discussion on the review topic were excluded, along with incomplete data, case reports, editorials, posters, and conference abstracts. Botanical names were verified using the web system “The Plant List” (https://powo.science.kew.org/) and ‘Plants of the World’ (http://www.theplantlist.org/). The structural formulae of the active metabolites were drawn using Chem Draw Ultra 15.0 software. This review adhered to the criteria outlined in the Preferred Reporting Items for Systematic Reviews and Meta-Analyses (PRISMA) statement (Page et al., 2021).

## 3 Mechanisms of MI

### 3.1 Pathogenesis of MI

Most instances of MI stem from the gradual progression of AS, primarily linked to endothelial dysfunction. Endothelial impairment increases the expression of adhesion molecules, attracting circulating immune cells, particularly monocytes, to the endothelial surface. These cells infiltrate the sub-endothelium and differentiate into macrophages (Libby et al., 2011). Macrophages engulf oxidized low-density lipoproteins beneath the endothelium, converting them into foam cells and contributing to the formation of a necrotic core. The expansion of this necrotic core, coupled with neoangiogenesis, can cause the rupture and bleeding of unstable plaques, triggering an intravascular thrombus. This series of events leads to persistent arterial constriction and eventual blockage of the vessel lumen (Tian and Fu, 2019). Consequently, a significant number of cardiomyocytes die due to severe coronary blood flow impairment. Even after blood reperfusion is restored, dysfunctional energy metabolism may persist in myocardial tissues. This is exacerbated by the accumulation of oxygen free radicals induced by ischemia and hypoxia, along with excessive intracellular Ca^2+^ levels in myocytes and subsequent inflammatory cascade responses. These factors contribute to mitochondrial dysfunction, worsening myocardial damage, and potentially resulting in serious complications such as malignant cardiac arrhythmias, myocardial fibrosis, and HF (Xue, 2019).

**FIGURE 1:**
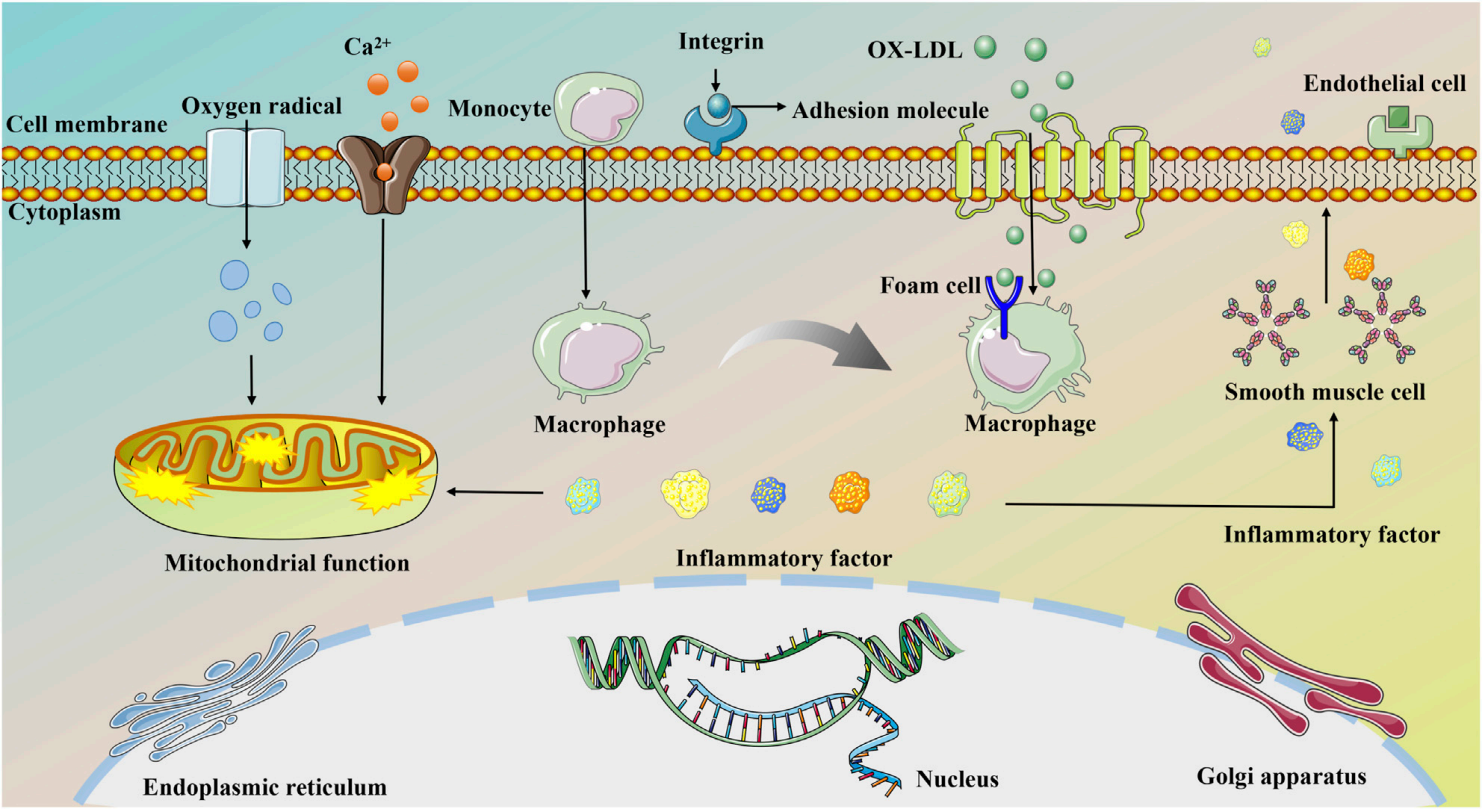
The pathogenesis of MI.

MI induces a state of myocardial tissue shock, characterized by reduced systolic and diastolic function and increased ventricular wall tension. This condition prompts the generation and release of NT-proBNP (Verhaar et al., 2022). Current studies suggest that structural alterations and malfunctions in the cardiac microcirculation resulting from endothelial injury, embolism, and microthrombosis are key factors contributing to inadequate ventricular perfusion post-PCI. The state of the microcirculation is intricately connected to endothelial function, with ET-1, a substance secreted by vascular endothelial cells to regulate vascular homeostasis, serving as an indicator of this function (Li et al., 2021). Figure 1 illustrates the pathogenesis of MI.

### 3.2 Molecular mechanisms of MI

**FIGURE 2:**
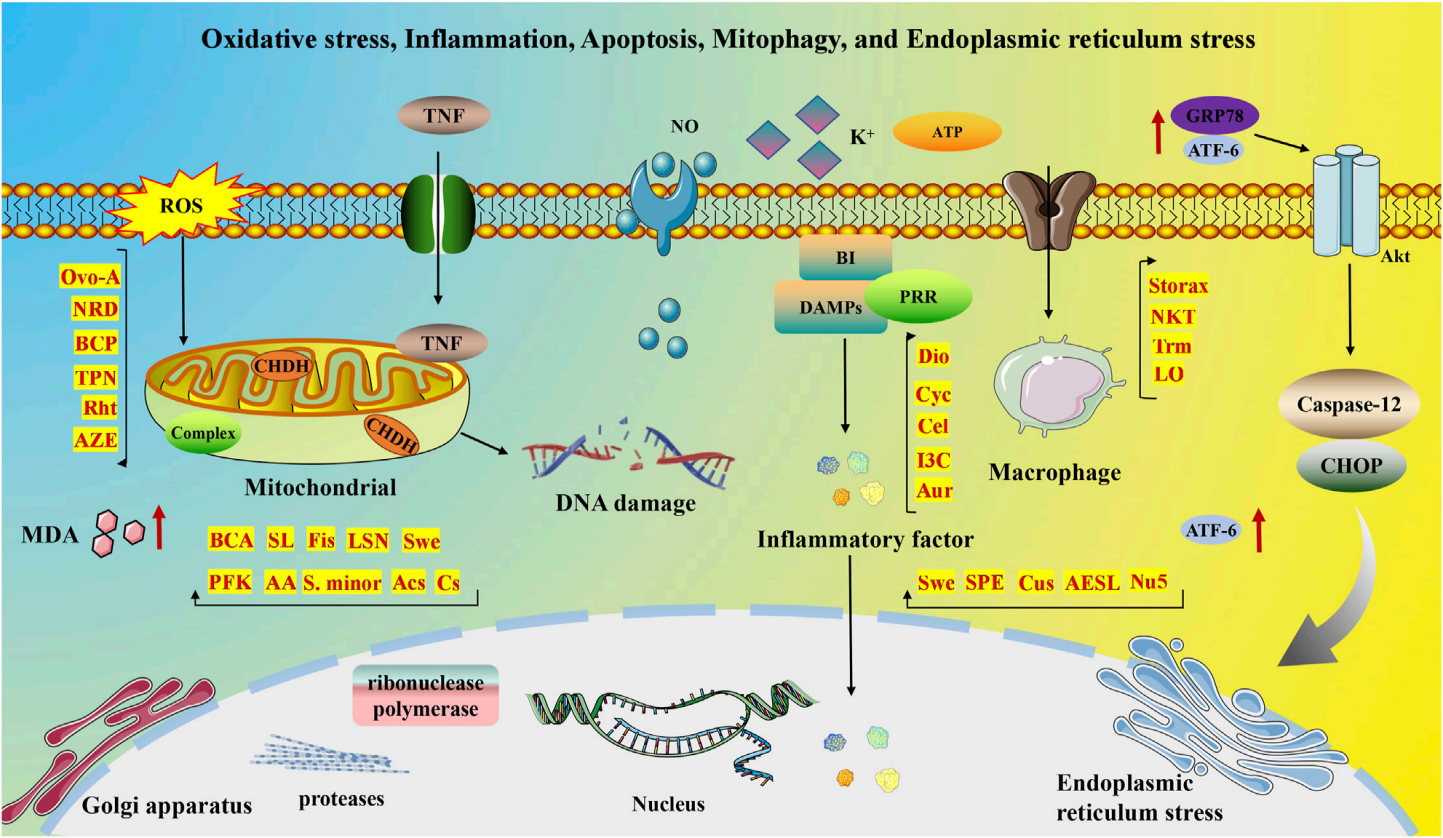
Molecular mechanisms of MI associated with oxidative stress, inflammation, apoptosis, mitophagy, and endoplasmic reticulum stress.

**FIGURE 3:**
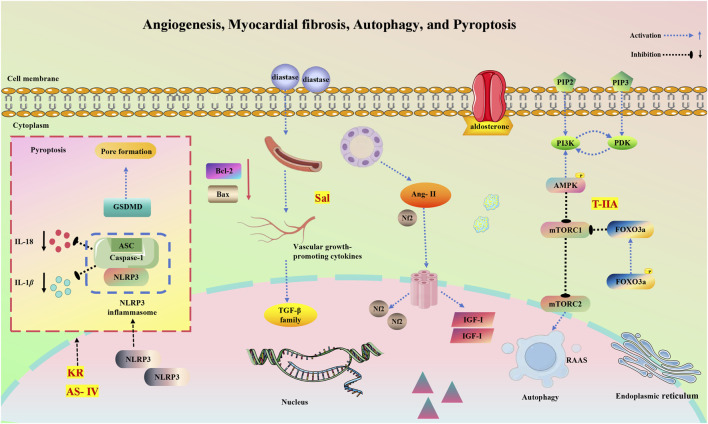
Molecular mechanisms of MI associated with angiogenesis, myocardial fibrosis, autophagy, and pyroptosis.

Despite significant advances in MI treatment, it remains a global disease with high morbidity and mortality. Although an ideal cure has not yet been found, therapeutic strategies focused on cardioprotection and post-ischemic cardiac repair are emerging. The molecular mechanisms underlying MI are significantly associated with inflammation, oxidative stress, apoptosis, neovascularization, myocardial fibrosis, autophagy, endoplasmic reticulum stress, mitophagy, and pyroptosis. This section delves into the specific mechanisms of MI from a molecular biology perspective. Figures 2, 3 illustrate the molecular mechanisms of MI.

#### 3.2.1 Oxidative stress and MI

The significance of oxidative stress and inflammation in the pathophysiology of MI and HF is well-documented. In a healthy heart, reactive oxygen species (ROS) function as signaling molecules, but their excessive and unregulated production results in oxidative stress and cardiomyocyte damage. This imbalance impairs mitochondrial function and increases ROS generation, triggering systemic inflammation and further damaging cellular components such as mitochondria. Consequently, this amplifies free radical production and intracellular oxidative stress, creating a detrimental cycle that accelerates MI progression (Cai et al., 2023). Moreover, elevated ROS levels in dysfunctional cardiomyocytes cause significant oxidative DNA damage, activating ribonuclease polymerase. The overactivity of this enzyme disrupts various cellular metabolic pathways and promotes the release of inflammatory mediators essential for cardiac remodeling and eventual failure (Yang et al., 2018).

MI is characterized by a systemic pro-inflammatory state. Excessive TNF expression damages mitochondrial DNA, impairs antioxidant factors, and disrupts mitochondrial complex III activity, leading to increased ROS production. Conversely, animal models of HF show that TNF inhibition stabilizes oxidative imbalance and reduces apoptosis (Tian et al., 2020). Additionally, oxidative stress catalyzes MI, with ROS causing structural and functional harm to cardiomyocytes (Yao et al., 2022). Patients with MI exhibit elevated serum nitrite levels, indicating stable NO breakdown. Studies reveal that patients with MI have increased serum MDA levels, elevated plasma oxidative stress markers, and reduced plasma antioxidant markers compared to healthy individuals. The oxidative stress process generates various complex compounds that disrupt normal cardiomyocyte function (Duan et al., 2023). Furthermore, oxidative stress-induced damage to cardiomyocytes manifests in several ways: it can damage proteins, lipids, nucleic acids, and other biomolecules, leading to abnormal cell function and death. It can disrupt calcium ion balance, increasing intracellular calcium levels, reducing cardiomyocyte contractility, and potentially triggering arrhythmias. Additionally, oxidative stress impairs mitochondrial function, reducing mitochondrial membrane potential, disrupting the mitochondrial respiratory chain, impairing energy metabolism, and leading to cell death.

#### 3.2.2 Inflammation and MI

In the context of MI, inflammation plays a dual role, aiding in the clearance of necrotic cellular debris during the initial phase and contributing to wound healing and scar formation during the reparative stage. Timely and appropriate suppression of inflammation can significantly reduce myocardial injury and enhance the healing process. Extensive research underscores the importance of early inflammatory activation for progressing toward later-stage healing and repair, necessitating coordinated cellular responses to promote wound healing and scar formation post-MI. These responses encompass three distinct yet interconnected phases: the inflammatory response, proliferation, and maturation (Frangogiannis, 2014). Post-MI, necrotic cardiomyocytes trigger an inflammatory reaction to eliminate dead cells and matrix remnants within the infarct area, laying the groundwork for mobilizing and stimulating mesenchymal repair cells. Substances released by necrotic cardiomyocytes, such as heat shock proteins and high mobility group box 1 histones, along with damaged extracellular matrix components, function as damage-associated molecular patterns (DAMPs). These DAMPs are recognized by pattern recognition receptors (PRRs) on white blood cells, activating the immune system and initiating subsequent inflammatory cascades.

Subsequent stages see significant upregulation of endothelial cell adhesion molecules, facilitating the extravasation and infiltration of circulating leukocytes from the bone marrow and spleen into the infarcted region. The infiltrating immune cells release various inflammatory mediators and proteases to clear damaged cells and debris from the extracellular matrix. Furthermore, the activation of the complement regulatory system and ROS production enhances the accumulation and clustering of immune cells in the infarct zone (Thackeray and Bengel, 2018). Following this, a proliferative phase ensues where the inflammatory response diminishes, and granulation tissue begins to form. Specific subgroups of monocytes and macrophages release substantial amounts of growth factors that stimulate fibroblast and endothelial cell proliferation. These cells also produce an abundant extracellular matrix, essential for maintaining the morphological and structural integrity of the left ventricle. Concurrently, they promote the development of new blood vessels to supply the wound with crucial oxygen and nutrients. This phase concludes with the apoptosis of repair cells, marked by the formation of a cross-linked collagen matrix network, granulation tissue cell apoptosis, and maturation of scar tissue (Oliveira et al., 2018).

#### 3.2.3 Apoptosis and MI

Apoptosis is recognized as a key mechanism through which cardiomyocytes undergo cell death in the context of MI (Li et al., 2016). During MI, myocardial cells suffer irreversible damage and necrosis due to hypoxia and a reduced supply of adenosine triphosphate (ATP). Necrotic cells subsequently release their contents, triggering immune system activation and a robust inflammatory response. The release of inflammatory mediators initiates tissue repair but can also lead to matrix degradation and apoptosis of cardiomyocytes. The inflammatory response typically persists for 0–4 days post-MI, during which various pathological changes occur among cells and numerous active substances are released. Ultrastructural changes during MI include myofibril relaxation, glycogen depletion, and mitochondrial swelling observed within a short period. With oxygen depletion, cellular damage becomes irreversible, and oxygen-deprived cells may undergo apoptosis, necrosis, or enter a state of “hibernation” (van den Akker et al., 2013). Ischemia-induced inflammatory response and oxidative stress can trigger apoptosis, and antioxidant therapies have demonstrated efficacy in reducing infarct size and mitigating subsequent HF (Desta et al., 2017). Research suggests that in MI, most cardiomyocytes perish through apoptosis, highlighting its role as a key determinant of post-infarction HF severity (Teringova and Tousek, 2017; Han et al., 2019). Recent findings indicate that apoptosis occurs not only within the infarcted region but also in cardiomyocytes both near and distant from the affected area. Molecular studies have explored the mechanisms of apoptosis in MI, revealing, for instance, that acetylation of FoxO3a can trigger Bim expression, furthering the progression of apoptosis (Xue et al., 2019).

#### 3.2.4 Angiogenesis and MI

Neovascularization refers to forming a new network of blood vessels based on the existing vascular system. In MI, neovascularization primarily invades the infarcted tissue from the ischemic border zone to restore oxygen and nutrient supply. Vascular endothelial cells detect and respond to neovascularization signals, initiating blood vessel formation with the sprouting of endothelial cells. These cells adhere to each other and connect to the extracellular matrix, which enzymes hydrolyze (Li et al., 2022). Subsequently, pericytes separate from the vessel wall, and connections between endothelial cells loosen, resulting in blood vessel dilation. Increased vascular permeability follows, causing plasma proteins to leak out and form a temporary extracellular matrix. Endothelial cells migrate through this matrix and differentiate into guiding apical cells or proliferating stalk cells. Stalk cells direct the outgrowth, proliferating to extend the conduit. Finally, a new lumen forms (Wu et al., 2018), encapsulating pericytes to ensure the new vessel’s stability and reintegration into circulation. Therapeutic neovascularization involves interventions that upregulate cytokines or receptors to promote new blood vessel growth in the ischemic myocardium. This process establishes collateral circulation, restoring blood supply to the ischemic myocardium and improving patient outcomes and prognosis. It is a key complementary strategy for treating MI and enhancing cardiac function. MI often begins with the rupture or erosion of atherosclerotic plaques, leading to the formation of superimposed thrombi. The resulting coronary artery occlusion damages the microcirculation, causing vessel disintegration within the infarcted area, capillary thinning, and a progressive reduction in cellular blood supply. Consequently, oxygen and nutrient transport, along with metabolic waste clearance, becomes restricted in the infarcted area (Wu et al., 2021).

Cardiomyocyte apoptosis and necrosis are pivotal in AMI progression. The loss of cardiomyocytes triggers pathological cardiac remodeling, significantly decreasing cardiac function and ultimately leading to HF (Zhu et al., 2021). Tissue repair following MI involves an angiogenic response from the border zone to the infarct core. Enhancing this response could serve as a safe and effective method for restoring blood supply to the infarcted area post-MI. Numerous factors influence neovascularization after MI, with various vascular growth-promoting factors playing a significant role. Post-MI, the expression of pro-vascular growth cytokines is upregulated in response to hypoxia, ischemia, inflammation, and other stimuli. These cytokines directly stimulate the proliferation and migration of mature endothelial cells within existing vasculature or indirectly prompt various cell types to increase the production of other pro-vascular growth factors.

#### 3.2.5 Myocardial fibrosis and MI

The activation of the RAAS system primarily drives myocardial fibrosis, exerting its biological effects by increasing both anterior and posterior cardiac loads through the release of angiotensin II (Ang II), aldosterone, and other bioactive substances. Ang II affects the myocardial interstitium by enhancing the permeability of coronary blood vessels, facilitating the entry of various pro-growth factors from the bloodstream, such as platelet-derived growth factor and basic fibroblast growth factor. This process leads to the proliferation and activation of cardiac fibroblasts, promoting their transition into myofibroblasts (Wang et al., 2017). Additionally, aldosterone upregulates the expression of tissue inhibitors of metalloproteinases-1 (TIMP-1) via the PI3K/NF-κB signaling pathway. TIMP-1, a widely distributed glycoprotein, disrupts the balance between collagen synthesis and degradation within the extracellular matrix by inhibiting the activity of matrix metalloproteinases (MMPs), specifically MMP-1, MMP-3, and MMP-9. Studies have shown a significant correlation between the imbalance of MMPs/TIMP-1/MMP inducers and infarct size, left ventricular function, and clinical prognosis following MI (Opstad et al., 2018).

Transforming growth factor TGF-β1 is the predominant active form closely linked to fibrosis in various organs, including the lungs, liver, and kidneys. Recent research has revealed that TGF-β stimulates a broad spectrum of cardiac fibroblast transcripts and promotes the secretion of myocardial exosomes via the Smad pathway, inducing MI phenotypes in cardiomyocytes (Maity et al., 2020), Under the influence of aldosterone, TGF-β can suppress MMP synthesis, enhance MMP production, and support cardiomyoblast survival. Galactaglutinin-3 (Gal-3), a beta-galactoside-binding lectin, is expressed in cardiac, renal, and adipose tissues, as well as in inflammatory cells. Gal-3 often acts as a downstream mediator of CT-1 in myocardial inflammation and fibrosis. Proteomic analyses have shown that after MI, CT-1 upregulates Gal-3 expression in cardiac fibroblasts along with other markers of fibrosis and inflammation (Martínez-Martínez et al., 2019). Gal-3 mediates the pro-inflammatory and fibrotic effects of CT-1, influencing the expression of extracellular matrix type 1 and atypical collagen. Insulin-like growth factor I (IGF-I) is a peptide with growth-promoting properties, found extensively in human tissues, including the liver, kidneys, lungs, heart, brain, and intestines. As a critical signaling molecule, IGF-I triggers the proliferation and differentiation of cardiac fibroblasts by activating various signaling pathways. Partial deletion and overexpression of IGF-I under defective conditions reduce myocardial contractility, alter sensitivity to Ang II, promote interstitial fibrosis, increase extracellular matrix collagen secretion, and change the expression patterns of genes related to cardiomyocyte calcium dynamics and cardiac structural proteins, confirming IGF-I’s role in myocardial fibrosis (González-Guerra et al., 2017). Hepatocyte growth factor (HGF), a versatile factor governing cell growth, motility, and morphogenesis, acts as an inhibitor of myocardial fibrosis. Its receptor is broadly expressed in the cardiovascular system, including cardiomyocytes, vascular endothelial cells, and smooth muscle cells. Through receptor phosphorylation and activation of downstream signaling pathways, HGF induces morphogenic, stem cell maintenance, and immunomodulatory effects. Liu et al. (2016) demonstrated that under hypoxic conditions, HGF significantly decreased the interaction between the anti-apoptotic protein Bcl-2 and the autophagy-associated protein Bcl-1. This interaction led to the sequestration of Bcl-2 by the pro-apoptotic protein Bax, effectively deactivating Bax and inhibiting cell apoptosis. Additionally, HGF enhanced the assembly of the autophagy-associated Beclin-1-VPS44-ATG14L complex, promoting cardiomyocyte autophagy and ameliorating cardiac remodeling following MI.

#### 3.2.6 Autophagy and MI

Endothelial cell dysfunction initiates atherosclerotic plaque formation, and the rapid progression of unstable plaques is crucial to MI development (Ahmadi et al., 2015). Unstable plaques are characterized by a thin fibrous cap and a large lipid core, composed mainly of vascular smooth muscle cells, macrophages (foam cells), collagen, and lipids. Studies indicate that defective endothelial autophagy promotes atherosclerosis, while upregulation of endothelial autophagy attenuates inflammatory injury and inhibits atherosclerosis progression (Vion et al., 2017). Additionally, macrophage and smooth muscle cell autophagy are closely linked to the progression of unstable plaques. Macrophages promote inflammation within the plaque and secrete matrix metalloproteinases, which degrade collagen and increase plaque rupture risk.

Inducing autophagy in peripheral blood monocytes via the AMPK-mTOR signaling pathway improves plaque stability, preventing rupture. Moreover, smooth muscle cell apoptosis results in fibrous cap thinning and increased lipid deposition, key features of unstable plaques (Cheng et al., 2015). Autophagy gene ATG7-deficient mice fed a high-fat diet exhibit increased smooth muscle cell apoptosis and exacerbated atherosclerotic instability compared to wild-type mice, suggesting that impaired smooth muscle cell autophagy may promote unstable plaque progression (Nahapetyan et al., 2019). MI leads to hypoxia, reduced ATP synthesis, increased lactic acid, and subsequent cardiomyocyte structural and functional damage (Aisa et al., 2017). Cardiomyocytes have limited differentiation and regeneration capacity; however, cellular autophagy can provide energy and stimulate self-renewal by breaking down dysfunctional organelles and proteins.

#### 3.2.7 Endoplasmic reticulum stress and MI

MI is a significant health concern. While interventional therapy has greatly increased the survival rate of patients with MI, ischemia/reperfusion (I/R) injury often occurs post-intervention, affecting the therapy’s overall efficacy. Studies have highlighted the critical role of endoplasmic reticulum stress and apoptosis in the pathological progression of I/R injury. During myocardial I/R, endoplasmic reticulum stress activates and upregulates GRP78, a key regulator. GRP78 stimulates the protein kinase B (AKT) signaling pathway, reducing ROS accumulation and mitigating cardiomyocyte injury (Bi et al., 2018). ATF-6, a downstream molecule of GRP78, also plays a protective role in MI and I/R pathology. Its activation reduces oxidative stress, regulates calcium homeostasis, and alleviates endoplasmic reticulum stress (Glembotski et al., 2019). The antioxidant effect of ATF-6 enhances the expression of proteins like catalase and inhibits the mitochondrial Ras system (Jin et al., 2017). Conversely, ATF-6 knockdown disrupts adrenergic insulin-like growth factor signaling, increasing mammalian target of rapamycin 1 (mTOR1) expression, inhibiting autophagy, and causing progressive myocardial hypertrophy (Blackwood et al., 2019).

Although moderate endoplasmic reticulum stress with GRP78 and ATF-6 activation is beneficial, the stress during MI and I/R tends to be persistent and severe. This severe stress, marked by the activation of CHOP and caspase-12, leads to excessive apoptosis and impaired cardiac function (Zhang et al., 2017). Therefore, inhibiting excessive endoplasmic reticulum stress is essential for alleviating I/R injury in MI treatment. Sodium 4-phenylbutyrate, a chemical chaperone, has been found to reduce endoplasmic reticulum stress, decrease cardiomyocyte apoptosis, minimize infarct size, and improve cardiac function (Takatori et al., 2017). Furthermore, in the endothelial cell hypoxia/reoxygenation assay, a considerable degree of endoplasmic reticulum stress was observed. Reoxygenation experiments revealed a notable elevation in the expression of GRP78, phosphorylated Caspase12, and CHOP, accompanied by a surge in apoptotic cell death. Conversely, acetylcholine demonstrated the capacity to activate the adenosine pathway. The AMPK pathway, via the type 3 muscarinic acetylcholine receptor (M3AChR), has been demonstrated to inhibit the apoptotic pathway associated with endoplasmic reticulum stress (Bi et al., 2015). Furthermore, it has been shown to protect the endoplasmic reticulum ultrastructure, thereby exerting a beneficial effect. The inhibition of excessive endoplasmic reticulum stress is beneficial to the treatment of MI. Recently, numerous studies have confirmed the close association between the mechanism of action of natural products and the amelioration of excessive endoplasmic reticulum stress.

#### 3.2.8 Mitophagy and MI

Mitophagy, a subtype of autophagy, degrades mitochondria through lysosomes in four key processes: initiation, elongation, maturation, and fusion. These stages are essential for autophagic vesicle formation and clearance, playing a vital role in intracellular mitochondrial quality control. During mitophagy, mitochondria are ubiquitinated and recognized by the ubiquitin receptor, facilitating degradation by binding to microtubule-associated protein 1A/1B-3 (MAP1LC3, LC3) on autophagosomes (Ji and Kwon, 2017). Research indicates that mitophagy is involved in the pathological mechanisms of cardiovascular conditions such as myocardial hypertrophy, MI, and HF (Wang et al., 2024). Studies using Beclin1^+^, FUNDC1 knockout, and transgenic mouse models suggest that mitophagy, rather than general autophagy, offers cardioprotection by regulating mitochondrial function (Xu C. et al., 2022a).

Asiatic acid regulates mitophagy energy metabolism through the AMPK pathway, alleviating mitochondrial edema, protecting ischemic cardiomyocytes, reducing infarct size and ischemic myocardial injury, and improving cardiac function (Qiu et al., 2022). The PINK1/parkin, FUNDC1, and Bcl-2/NIX pathways primarily regulate mitophagy. Additionally, autophagy receptors like OPA1 and NDP52, as downstream effectors of the PINK-Parkin signaling pathway, regulate mitophagy in the heart. Upon activation, choline dehydrogenase (CHDH) accumulates on the outer mitochondrial membrane (OMM), interacts with p62, and recruits’ autophagy adapters to depolarized mitochondria. The choline-p62-LC3 complex is involved in Parkin-mediated mitophagy. Tax1-binding protein 1 (TAX1BP1) regulates mitophagy by controlling mTOR (Whang et al., 2017). OPA1-mediated mitophagy protects cardiomyocytes from oxidative stress by suppressing mitochondrial division, essential for maintaining redox balance, and presents a promising target for future MI therapies (Whang et al., 2017; Wang Y. et al., 2021b).

#### 3.2.9 Pyroptosis and MI

Cellular pyroptosis, a form of programmed cell death regulated by Gasdermin, involves membrane pore formation leading to cell swelling, rupture, and the release of pro-inflammatory cytokines. These cytokines trigger and escalate inflammatory reactions, which are fundamental pathological features of CVD. The activation of pyroptosis and its associated inflammasomes is closely linked to the development of cardiovascular conditions (Zhou W. et al., 2018b). MI typically involves a sterile inflammatory response, with infarcted cardiomyocytes showing increased levels of NLRP3. Myocardial tissues from patients with MI display marked infiltration of inflammatory cells and high expression of ASC proteins (Kawaguchi et al., 2011). Silencing caspase-1 or NLRP3 in mice reduces mortality and attenuates the inflammatory response during MI (Sandanger et al., 2013). This indicates that caspase-1 and NLRP3-mediated pyroptosis significantly impact MI/RI (Luan et al., 2022a; Luan et al., 2022b). Following MI, cellular debris and metabolites act as DAMPs, activating inflammasomes and leading to a sterile inflammatory response.

In MI models, cardiomyocytes and fibroblasts (Sandanger et al., 2013) exhibit increased expression of pyroptosis markers, and inhibiting pyroptosis reduces infarct size, improves cardiac function and ventricular remodeling, and increases survival rates (Audia et al., 2018). Colchicine, a non-specific inhibitor of the NLRP3 inflammasome, significantly reduces infarct size and inflammatory markers, as demonstrated in a phase II clinical trial. This suggests that colchicine may improve MI outcomes by inhibiting pyroptosis (Deftereos et al., 2015). The onset of pyroptosis is associated with K^+^ efflux, lysosomal destabilization, and the production of ROS. Research shows that oxidative stress can exacerbate MI by triggering cardiomyocyte pyroptosis through the activation of nuclear factor κB (Lei et al., 2018).

Furthermore, recent studies (Mao et al., 2019) have revealed that lncRNA.KLF3-AS1 competitively binds to miR-138-5p, thereby inhibiting cardiomyocyte pyroptosis. Other non-coding RNAs, such as miR-135b (Li et al., 2020), can also impede MI progression by inhibiting the onset of cellular pyroptosis. This suggests that the prevention of cardiomyocyte death, both at the coding and non-coding levels, may enhance cardiac function.

## 4 Some lesions caused by MI

**FIGURE 4:**
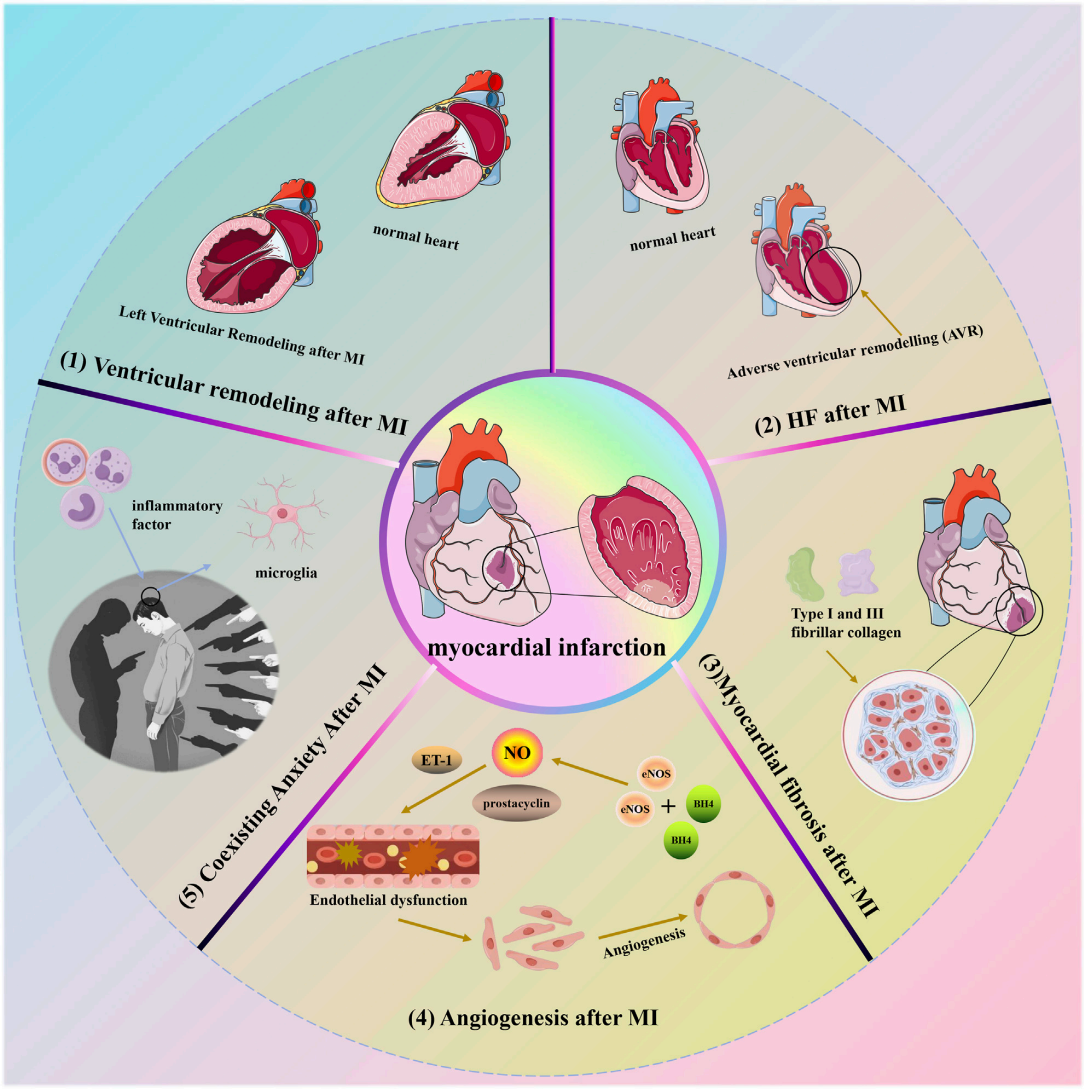
Some lesions caused by MI.

MI refers to extensive myocardial necrosis resulting from prolonged ischemia due to coronary blood flow interruption. Clinically, it presents with severe and persistent retrosternal pain that is not fully relieved by nitrate medications or rest. MI can also lead to additional complications such as ventricular remodeling, HF, myocardial fibrosis, angiogenesis, and potentially depression. Figure 4 illustrates some of the symptoms associated with cardiac infarction.

### 4.1 Ventricular remodeling (VR) after MI

MI is defined as tissue necrosis due to inadequate perfusion, resulting in cardiomyocyte death (Ibanez et al., 2018). This initiates a cascade of intracellular signaling reactions leading to molecular, cellular, and interstitial alterations, which manifest clinically as changes in the size, shape, and function of the heart. These complex changes characterize the phenomenon of cardiac remodeling. Initially, cardiac remodeling acts as an adaptive mechanism to maintain cardiac function. However, with prolonged persistence, it leads to chronic progressive ventricular dysfunction and eventual mortality (Azevedo et al., 2016). Importantly, MI induces structural and functional changes in the heart that exacerbate oxidative stress, inflammation, and collagen deposition, while also affecting energy metabolism. These changes are characteristic features of cardiac remodeling (Pereira et al., 2017; Oliveira et al., 2021). Current strategies aimed at preventing or mitigating post-MI remodeling include reperfusion therapy and advanced pharmacological interventions. Several medications, such as angiotensin-converting enzyme inhibitors, Ang II receptor blockers, beta-blockers, aldosterone antagonists, and agents targeting enkephalin lytic enzymes and angiotensin receptors, have proven effective in improving outcomes following MI (Ibanez et al., 2018). Recent studies indicate that attenuating cardiac remodeling after MI can be achieved by reducing fibrosis and hypertrophy while enhancing oxidative stress, and energy metabolism, and mitigating inflammatory responses (Pereira et al., 2020).

### 4.2 Heart failure (HF) after MI

Numerous medications have demonstrated efficacy in improving outcomes following MI, such as angiotensin-converting enzyme inhibitors, Ang II receptor blockers, beta-blockers, aldosterone antagonists, and agents targeting enkephalin lytic enzymes and angiotensin receptors (Virani et al., 2020; Tsao et al., 2023). HF subsequent to MI results from structural and functional abnormalities that arise post-AMI, encompassing both ST-segment elevation MI (STEMI) and non-ST-segment elevation MI (non-STEMI). These changes in cardiac structure and function are collectively known as adverse ventricular remodeling (AVR). HF following MI typically involves AVR in the left ventricle (LV), potentially due to its higher stroke volume compared to the right ventricle (RV), which primarily supports cardiac pumping. Post-MI AVR involves adjustments in ventricular dimensions, morphology, and performance, influenced by regulatory factors such as neurohormones, inflammation, energy metabolism, and genetic predispositions (Bhatt et al., 2017; Swirski and Nahrendorf, 2018). Additionally, the pathophysiological mechanisms contributing to HF may be complex, not solely attributed to left ventricular systolic dysfunction and volume overload but also potentially linked to right ventricular impairment or HF with preserved ejection fraction (HFpEF) (Del Buono et al., 2023). Furthermore, a cascade of effective compensatory responses initiated by right ventricular infarction may augment the prospects for ischemic myocardial recovery (Nägele and Flammer, 2022). Nevertheless, despite significant strides in elucidating the underlying mechanisms of HF post-MI, several questions remain unanswered. Consequently, further investigations focusing on the interaction between these diverse mechanisms are crucial to achieve a comprehensive understanding of HF complexities following MI.

### 4.3 Myocardial fibrosis after MI

Cardiac fibrosis is characterized by an excess of actively dividing cardiac fibroblasts, resulting in thickening or scarring of heart tissue that distorts and impairs its structural and mechanical functions (Yan et al., 2022). This condition serves as a critical pathological indicator of cardiac remodeling and contributes to cardiac dysfunction (Cai et al., 2017). These fibroblasts produce type 1 and type 3 fibrillar collagen, the primary components of the myocardial interstitium. Type 1 collagen forms thick fibers with lower tensile strength and elasticity, while type 3 collagen forms a delicate network more easily stretched than type 1 collagen (Snider et al., 2021). An increase in overall myocardial collagen content or a shift in the ratio of collagen types 1 and 3 can disrupt the typical myocardial fiber network structure. This disruption restricts normal cardiomyocyte expansion and contraction, leading to reduced myocardial compliance, ejection capacity, increased myocardial stiffness, and impaired diastolic or systolic myocardial function (Lai et al., 2022). Following AMI, left ventricular remodeling often occurs, compromising coronary blood flow, increasing myocardial fibrosis, and reducing cardiac function. Consequently, patients typically observe minimal advancements in clinical symptoms and prognosis (Jiao et al., 2021). Moreover, apoptosis significantly contributes to the development of post-MI cardiac insufficiency and structural changes in the myocardium, leading to myocardial fibrosis and eventually symptomatic HF (Goodman et al., 2020). Therefore, prioritizing anti-apoptotic mechanisms is essential to combat myocardial fibrosis and preserve cardiac function. Increasing evidence suggests that activation of the renin-angiotensin-aldosterone system (RAAS), which triggers apoptosis, plays a pivotal role in the pathogenesis of myocardial fibrosis (Spoladore et al., 2021). Thus, interventions aimed at targeting the RAAS pathway, such as Ang II type 1 receptor blockade, have shown effectiveness in modern clinical practices for managing myocardial fibrosis and improving myocardial function (Niu et al., 2020).

### 4.4 Angiogenesis after MI

The widely distributed vascular endothelium plays a vital role in regulating vascular tone through the production and release of various endothelium-derived factors that promote vasodilation, including NO, prostacyclin, and endothelium-derived hyperpolarizing factors (Godo and Shimokawa, 2017). The impairment of vascular endothelial function results from an imbalance in vasoactive substances and cytokines released by compromised endothelial cells (Mohr et al., 2017). Endothelial dysfunction is characterized by reduced bioavailability of NO, which may adversely affect long-term remodeling processes (Klinger et al., 2013). Under normal physiological conditions, endothelial cell nitric oxide synthase (eNOS), working with the redox-sensitive cofactor tetrahydrobiopterin, plays a crucial role in NO synthesis. Nitric oxide serves as a signaling molecule contributing to vascular homeostasis and potentially influencing cardiomyocyte function (Edgar et al., 2017). Nevertheless, vascular remodeling following MI leads to the oxidation of BH4, which triggers the production of uncoupled eNOS-derived superoxide, exacerbating the progression of remodeling and impairing cardiac function (Hashimoto et al., 2016). Additionally, endothelin 1, a vasoconstrictor peptide synthesized by the endothelium, plays a crucial role in maintaining vascular tone in healthy individuals. However, after MI, there is an increase in the expression and levels of endothelin receptor A, further intensifying its effects (Chen et al., 2016). The renin-angiotensin system (RAS) is pivotal in the onset and development of various CVD. Activation of the RAS leads to elevated levels of Ang II, which impairs eNOS function and raises ET-1 levels (Maneesai et al., 2016). Research indicates that the progression of endothelial dysfunction and the exacerbation of MI can be linked not only to the presence of nitric oxide but also to an imbalance between eNOS-derived nitric oxide and ET-1 (Zhou et al., 2014).

### 4.5 Coexisting anxiety after MI

The inflammatory response acts as a common pathological mechanism in both MI and anxiety. After MI, chronic stress worsens the inflammatory state by persistently activating sympathetic nerves, diminishing vagal tone, and upsetting the autonomic nervous system’s internal equilibrium. These alterations affect neurotransmitter regulation, ultimately influencing anxiety-related behaviors following MI (Steptoe et al., 2013). In addition, inflammatory factors in the bloodstream can regulate microglial activation and impact the synthesis of monoamine neurotransmitters either by compromising the blood-brain barrier or by directly infiltrating the brain parenchyma (Wang et al., 2018a). Research has also shown that peripheral inflammation ultimately alters neural circuitry in the brain, influencing regions sensitive to reward and threat, and triggering anxiety-like behavior (Felger, 2018).

## 5 Methods for developing animal models of MI

**FIGURE 5:**
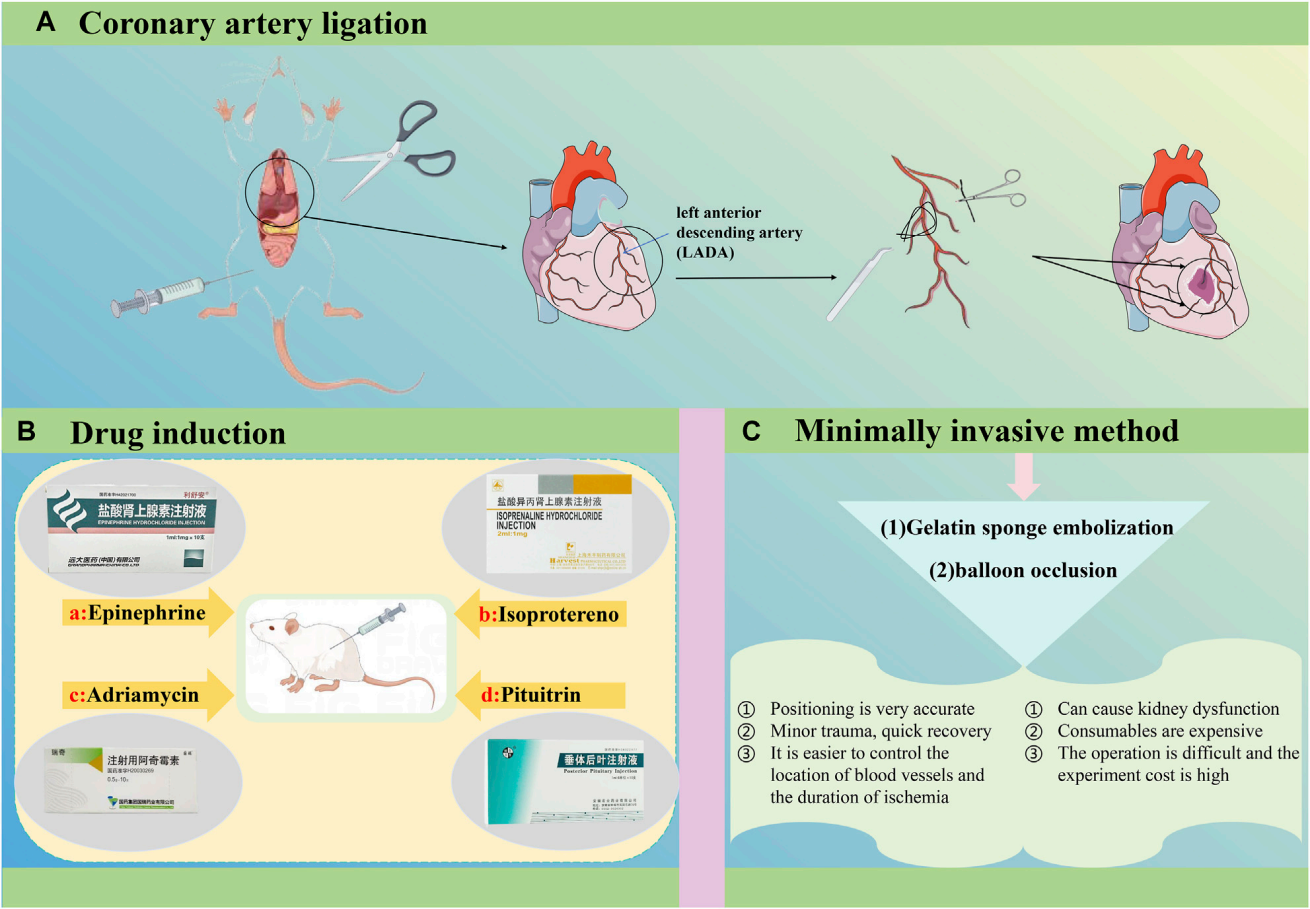
Three commonly used methods for establishing animal models of MI are coronary ligation, pharmacological induction, and minimally invasive methods.

There are several methods available to establish animal models of MI, such as coronary ligation, drug induction, and minimally invasive techniques (Tang et al., 2018). Currently, rabbits, minipigs, and rats are the most chosen animals for MI modeling in laboratory research (Figure 5).

### 5.1 Coronary ligation

Currently, coronary ligation is the preferred method for modeling MI (Williams et al., 2013). In animal models, MI is frequently induced by ligating the left anterior descending branch of the coronary artery. This ligation results in stenosis or occlusion of the artery, causing ischemia and necrosis in the myocardial region it supplies, effectively simulating MI in the animal model (Wang et al., 2020). Specific procedures also involve abdominal anesthesia combined with mechanical ventilation and cardiac compression (Gao et al., 2010). The former involves opening the chest cavity to induce MI under the microscope, a procedure that is both traumatic and time-consuming. The latter method, introduced by Gao et al. (2010), involves artificial exposure through a small incision without intubation and is widely adopted due to its advantages of speed, minimal trauma, and low surgical mortality (Snider et al., 2021; Gan et al., 2022). Coronary artery ligation is well-established and straightforward to perform, providing a clearly identifiable site of obstruction that aligns closely with the pathological process of MI. It facilitates clinical translation and offers high flexibility in modeling, allowing real-time monitoring and evaluation through ECG, pathology, and serum enzymology.

### 5.2 Pharmacological methods

Commonly used drugs currently include epinephrine, isoproterenol (ISO), Adriamycin (Fu et al., 2021), and pituitrin (Lin et al., 2017), among others. These drugs primarily induce MI by causing spasms in the coronary artery, with ISO being the most widely employed (Lobo Filho et al., 2011). It's important to note that the drug-induced method of creating an MI model is generally considered straightforward, requiring relatively low technical expertise. This method effectively mimics the vasoconstriction process observed during human MI onset. However, it poses challenges in controlling the extent of MI, specifying the affected artery, and achieving consistent outcomes.

ISO, an artificial catecholamine, plays a crucial role in regulating myocardial contraction and metabolism, serving as a standard model for studying the effects of drugs on cardiac function (Saravanan et al., 2013). ISO-induced MI has been extensively studied in animal experiments, closely resembling human conditions (Huang et al., 2018). This model results in myocardial necrosis characterized by increased oxygen consumption, impaired oxygen utilization, calcium overload, altered membrane permeability, intracellular acidosis, and elevated lipid peroxidation. Such necrosis typically arises from coronary artery obstruction, often due to an atherosclerotic clot or arterial spasm (Slavich and Patel, 2016). ISO is employed to induce MI models for evaluating the cardioprotective effects of various agents; doses as high as 100 mg/kg can induce necrosis, hypoxia, and pathological changes typical of MI (Lobo Filho et al., 2011). Additionally, adrenaline-induced MI in rats has been recognized as a reliable experimental model for studying the antioxidant’s cardioprotective effects (Bhattacharjee et al., 2019).

### 5.3 Minimally invasive methods

Minimally invasive techniques primarily encompass gelatin sponge embolization (Sun et al., 2018) and balloon occlusion (Kőrösi et al., 2023), among others. These methods exhibit the following characteristics: firstly, they enable precise selection of any coronary artery under contrast guidance, ensuring accurate positioning. Secondly, the minimally invasive approach results in minimal surgical trauma, thereby preserving the internal chest cavity environment and causing minimal impact on other vital organs, allowing experimental animals to recover quickly post-operation. Lastly, balloon occlusion facilitates precise control over the location and duration of coronary artery ischemia, which is beneficial for studying myocardial reperfusion injury. However, this method also presents certain drawbacks: firstly, the contrast agent used can induce abnormal renal function in animal models. Secondly, it necessitates angiographic equipment, skilled operators, and costly interventional supplies. Lastly, operating on small animals like rabbits and rats is more challenging due to their narrower arterial diameter, leading to increased experimental costs associated with selecting larger animals.

## 6 Natural products for targeted treatment of MI

### 6.1 Natural metabolites targeted for the treatment of MI

**FIGURE 6:**
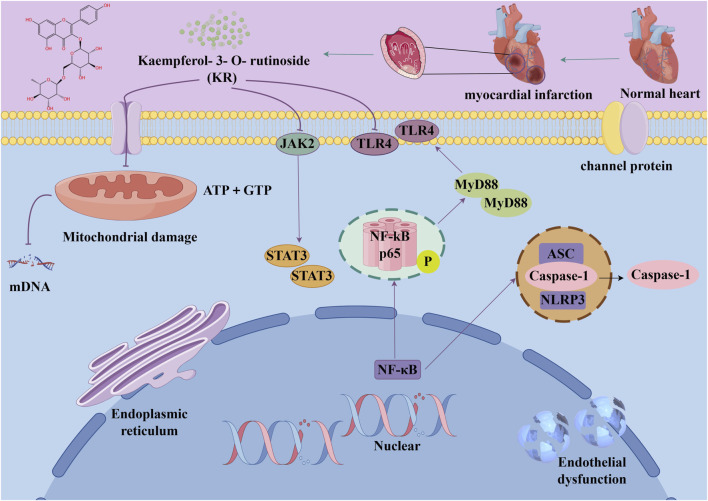
The example of kaempferol-3-O-rutinoside provides a detailed illustration of the potential benefits of natural products in the treatment of MI, which can be attributed to their capacity to target multiple pathways and mechanisms.

Several recent studies confirm that natural active metabolites exhibit a broad spectrum of pharmacological activities. There is an increasing inclination towards employing these natural metabolites as complementary strategies in the prevention and treatment of MI. This article provides a comprehensive summary of 20 natural active metabolites directly linked to MI within the past 5 years. We have categorized and analyzed their primary bioactive metabolites (Table 1) and illustrated in Figures 2, 3 the molecular mechanisms through which various natural active metabolites from medicinal plants contribute to MI treatment. Figure 6 specifically highlights how natural products, exemplified by kaempferol-3-O-rutinoside, target multiple pathways and mechanisms in the treatment of MI.

**TABLE 1:** Natural active metabolites for the treatment of MI from 2018 to 2023.

No	Natural products	Chemical structure	Types	Dosages	Models	Effects/Mechanisms	References
1	Ovothiol-A	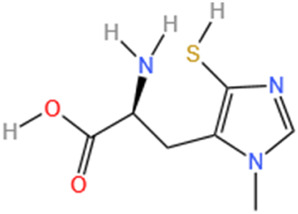	*In vivo*	500 mg/kg i.g., every day for 7 days	Epinephrine induced MI model	Cardiac troponin T, creatine kinase, LDH, and AST levels ↓; ALT and ALP levels ↓; TP levels ↑; creatinine, uric acid, and urea concentration ↓; MDA and NO levels ↓; GSH, CAT, and GST activities ↑; hemoglobin, red blood cells, white blood cells, PLR, and NLR levels ↓	Mohammed et al. (2022)
2	Nerolidol	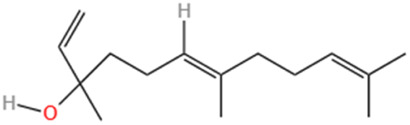	*In vivo*	50 and 100 mg/kg i.g.	ISO induced MI model	LVDP, +dp/dt, and -dp/dt ↓; CK, CK-MB, and LDH levels ↓; infarct size ↓; SOD activity ↑	Gonçalves et al. (2022)
3	Salvianolate	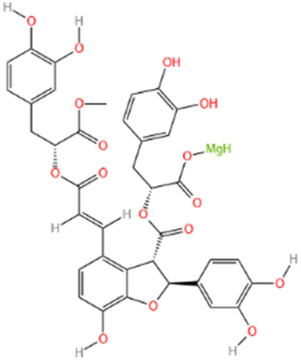	*In vivo*	24.219 and 48.438 mg/kg i.g., every day for 28 days	LAD induced MI model	EF and FS levels ↑; infarct size ↓	Chen et al. (2022)
			*In vitro*	0, 1, 2, 4, 8, and 16 μmol/L incubated for 24 h	—	CaN activity ↓; the mRNA levels of b-MHC and CaNA ↓; NFA Tc3 level ↓	
4	β-caryophyllene	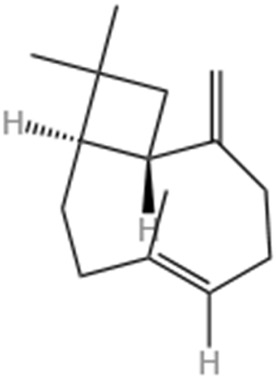	*In vivo*	50 mg/kg i.g., twice a day for 7 days	ISO induced MI model	CB2 receptor and PPAR levels ↓; GRK2 and β-catenin protein levels ↑; troponin-T, CK, and LDH levels ↑; infarct size ↓; TBARS and LOOH levels ↓; SOD, catalase, and GSH activities ↑; p-PI3K, p-Akt, HO-1, and Nrf2 protein levels ↑; Keap-1 protein level ↓; TBARS level ↓; TNF-α, IL-1β, and IL-6 levels ↓; expression of HMGB1, iNOS, and COX-2 ↓; NLRP3, caspase-1- p20, procaspase-1, pro-IL-1β, TLR4/NF-κB, and MAPK protein levels ↓; P -mTOR protein level ↑; total cholesterol, triglycerides, free fatty acids, free cholesterol, and cholesterol ester levels ↑; serum and cardiac phospholipid levels ↓; HMG-CoA reductase activity ↓; LCAT activity ↑; ATPase and electrolyte levels ↓	Meeran et al. (2021b)
5	Alpha-terpineol	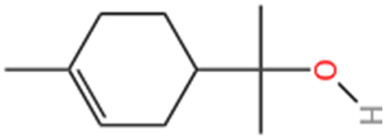	*In vivo*	25, 50, and 75 mg/kg i.g., every day for 15 days	ISO induced MI model	Collagen deposition area ↓; CKT and CKMB activities ↓; reactive C protein level ↓; calcium levels ↑; AHW, Wh/Wb, Wh/Wf, RHW, and ΔTLV ↓; rat survival rate ↑	Paulino et al. (2022)
6	Diosmetin	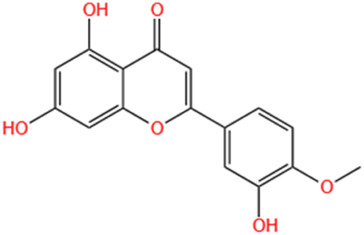	*In vivo*	1 and 3 mg/kg i.g., every day for 6 days	ISO induced MI model	Body/weight ↓; cTnI, CPK, CK-MB, LDH, AST, and ALT levels ↓	Ahmad et al. (2023)
7	Kaempferol-3-O-rutinoside	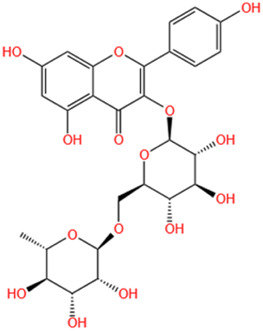	*In vivo*	10 mg/kg i.g., every day for 28 days	LAD induced MI model	LVSP, +dp/dt max, and -dp/dt max levels ↓; LVEDP ↑; collagen levels ↓; CVF% ↓; mRNA expression of NLRP3, caspase-1, GSDMD, NF-κB p65, and IL- 1β ↓	Hua et al. (2022)
8	Biochanin-A	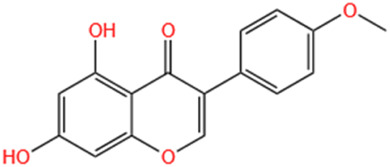	*In vivo*	10 mg/kg i.g., every day for 30 days	ISO induced MI model	MDA content ↓; cTnI level ↓; SOD and CAT activities ↑; GPX, GST, GSH, and GRD levels ↑	Govindasami et al. (2020)
9	Cycloastragenol	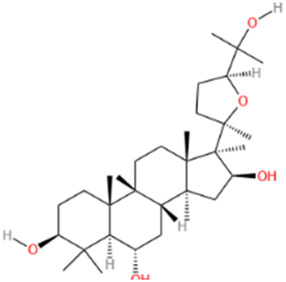	*In vivo*	5, 10, and 20 mg/kg i.g., every day for 35 days	LAD induced MI model	Caspase-9, caspase-3, and Bax ↓; AKT, ERK, and NF-κB-p65 ↓; PLCG1, RhoA, and GSK-3β expression ↓; apoptosis cell ↓; RhoA and AKT expression ↑; EF, FS, PWTd, and PWTs ↑; LVIDs and LVIDd ↓; SBP and DBP levels ↑; TNF-α and IFN-γ ↓; IL-4 levels ↑; IL-1β, IL-6, IL-8, and IL-17 levels ↓; IL-10 levels ↑	Ren et al. (2020)
10	S-limonene	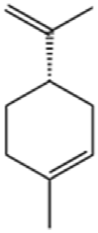	*In vivo*	0.1, 1, 3, 10, and 30 mg/kg i.g	ISO induced MI model	Infarct size ↓; CK-total and CK-MB ↓; inflammatory cell infiltrate, fibrotic area, and total collagen ↓; ST-segment, heart rate, and QTc interval ↓; Ca^2+^ ↓; mitochondrial density and ROS generation ↓; enzymatic activity of GPX, total SOD, Mn-SOD, and Cu–Zn-SOD activities ↑; LVDP and +dp/dt ↑; arrhythmia score ↓	Rhana et al. (2022)
11	Celastrol	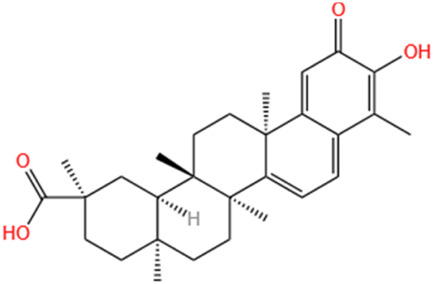	*In vivo*	1 mg/kg i.g., every other day for 28 days	LAD induced MI model	LVEF, LVFS, LVESP, +dp/dt max, and -dp/dt max ↑; collagen I, collagen III, and α-SMA levels ↓; IL-18 and IL-1β levels ↓; inflammatory cell infiltration ↓; NLRP3 activation ↓; cleaved caspase-1 ↓	Fan et al. (2023)
12	Fisetin	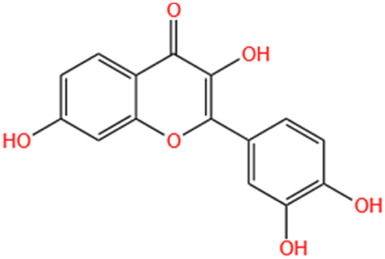	*In vivo*	20 mg/kg i.g., every day for 28 days	LAD induced MI model	Infarct size ↓; heart rate and SAP & DAP ↑; SOD, catalase, and GSH levels ↑; MDA level ↓; LDH and CK-MB levels ↓; TNF-α and IL-6 levels ↓; edema, inflammatory cell infiltration and myonecrosis ↓; Bcl2 & PPAR-γ level ↑; caspase-3 and Bax ↓; NF-κB-p65 expression ↓; p-ERK ↑; p-p38 and p-JNK ↓	Garg et al. (2020)
13	Indole-3-carbinol	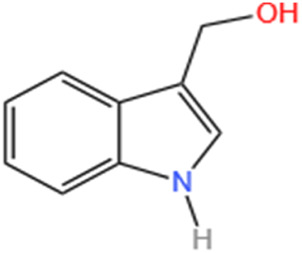	*In vivo*	—	ISO induced MI model	The prolongation of the QRS complex, QT interval, and ST-segment elevation ↓; heart weight, cTnI, CK-MB, LDH, AST, and ALT ↓; SOD, CAT, and GSH levels ↑; MDA level ↓; TNF-α and IL-6 levels ↓; 1L-10 level ↑; neutrophil infiltration and oedema ↓; cytochrome C, caspase 9, and caspase 3 ↓	Ramakrishna and Krishnamurthy (2022)
14	Nootkatone	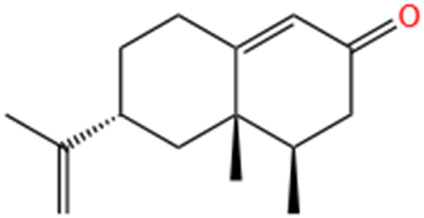	*In vivo*	10 mg/kg i.g., every day for 11 days	ISO induced MI model	Serum levels of CK, LDH, and troponin-T ↓; heart rate, SP, DP, and MAP ↑; TBARS and LOOH levels ↓; SOD, catalase, GSH, vitamin-C, and vitamin-E contents ↑; p-PI3K, p-Akt, HO-1, and Nrf2 proteins levels ↑; Keap-1 expression ↓; β-glucuronidase, β-galactosidase, cathepsin-B, and cathepsin-D activities ↓; TNF-α, IL-6, and IL-1β levels ↓; HMGB1, iNOS, and COX-2 expression ↓; TLR4, p-NF-κB-p65, p-IκBα, p-ERK1/2, p-P38, and p-JNK proteins levels ↓; Bax, cytochrome-C, cleaved caspase-9, and cleaved caspase-3 proteins levels ↓; Bcl-2 and Bcl-xL proteins levels ↑; Na^+^/K^+^ and ATPase ↑; Ca^2+^ and Mg^2+^ ATPases activities↓	Meeran et al. (2021a)
15	Liensinine	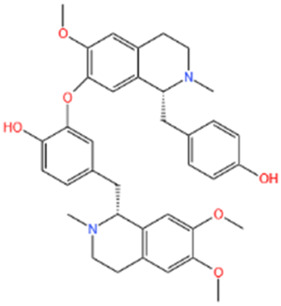	*In vivo*	10 mg/kg i.g., every day for 28 days	LAD induced MI model	LV, EF, and FS parameters ↑; CTNI, CTNT, ANP, and BNP levels ↓; infarct size ↓; cardiac fibrosis markers ↓; γ-H2AX ↓	Shen et al. (2023)
			*In vitro*	0.5 µM induced for 24 h		γ-H2AX ↓; β-catenin ↓; Wnt/β-catenin pathway ↓	
16	Auraptene	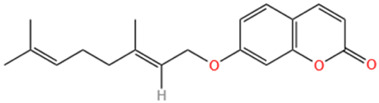	*In vivo*	5 and 50 mg/kg, i.g., every day for 42 days	LAD induced MI model	ANF and ET-1 promoter activation ↓; PPARα ↑; glutathione S-transferase activity ↑; LVEDD, LVESD, and PWT ↓; LVFS% ↑; myocardial cell diameter ↓; ANF, BNP, and ET-1 transcription levels ↓; perivascular fibrosis ↓; MCP-1 and collagen III proteins levels ↓; MFN2, transcription factor A, TFAM, MCAD, and mCPT1 levels ↑	Sunagawa et al. (2022)
17	Tanshinone IIA	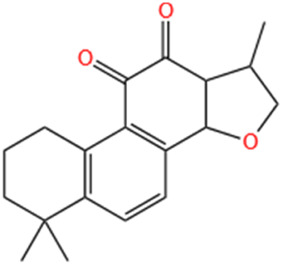	*In vivo*	1.5 mg/kg, i.g., every day for 28 days	LAD induced MI model	EF% and FS% ↑; LVIDs and LVIDd ↓; Bcl-2 and Bax expression ↓; cleaved caspase-3 and cleaved caspase-7 expression ↓; LC3, Beclin1, ATG3, and ATG7 transcript expression ↑; p62 expression ↓; P-AMPKT 172 expression ↑; P-mtorser2448 and P-ulk1ser 757 expression ↓; S6K1 expression ↓; AMPK and LC3 expression ↑; phosphorylated mTOR, p62, and S6K1 expression ↓	Zhang et al. (2019)
18	Swertiamarin	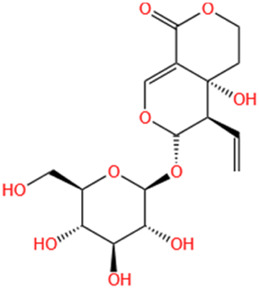	*In vivo*	20 and 40 mg/kg i.g., every day for 7 days	ISO induced MI model	Body weight ↑; heart weight/tibia length ↓; CK-MB, LDH, AST, ALT, and cTn1 levels ↓; MDA and PC levels ↓; Vitamin C and Vitamin E levels ↑; GSH, SOD, CAT, GPX, GST, GR, and TAC levels ↑; Na^+^/K^+^ potassium and Ca^2+^ ATPase levels ↑; TNF-α and IL-6 levels ↓	Wang et al. (2023)
19	Astragaloside IV	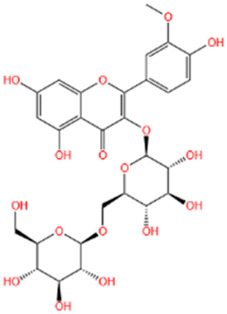	*In vivo*	25 and 50 mg/kg, i.g., every day for 14 days	LAD induced MI model	EF% and FS% ↑; LVIDd and LVIDd ↓; infarction area ↓; rat survival rate ↑; collagen content ↓; Bax protein expression ↓; Bcl-2 protein expression ↑; VEGF expression ↑; PTEN expression ↓; PI3K and phosphorylated Akt levels ↑	Cheng et al. (2019)
20	Curcumin	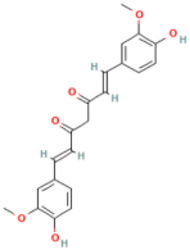	*In vivo*	150 and 200 mg/kg Cs and 100, 150, and 200 mg/kg nC	ISO induced MI model	R-R interval ↑; heart rate ↓; expansion QRS complex ↓; QT interval lengthening ↓; ST suppression and T wave inversion ↓; LDH, AST, and ALT levels ↓; MDA content ↓; mercaptan and TAC levels ↑	Boarescu et al. (2019)

#### 6.1.1 Astragaloside IV (AS-IV)

AS-IV, a traditional Chinese herb with a legacy spanning over 2000 years, contains AS-IV as its primary extracted monomer (Ren et al., 2013). Prior research has indicated that AS-IV exhibits various pharmacological activities, including antioxidative stress, anti-inflammatory properties, apoptosis inhibition, and promotion of energy metabolism, among others. Consequently, it is extensively utilized in the treatment of CVD (Zhang et al., 2018). Administered via gavage for 14 days at doses of 20 and 50 mg/kg, AS-IV significantly reduced collagen content and improved myocardial fibrosis in a rat model of MI. Moreover, AS-IV suppressed PTEN to promote angiogenesis through activation of the PTEN/PI3K/AKT signaling pathway downstream (Cheng et al., 2019). It has also been found that AS-IV ameliorates adverse ventricular remodeling, as well as attenuates myocardial fibrosis and cardiac hypertrophy by decreasing NLRP3/caspase-1/GSDMD signaling pathway protein expression. *In vitro* experiments suggest that the cardioprotective effects of AS-IV may be related to the reduction of macrophage pyroptosis (Zhang X. et al., 2022b).

#### 6.1.2 Curcumin (Cs)

Cs, a nutraceutical metabolite known for its strong antioxidant activity, is a promising candidate for combating oxidative stress-induced damage. Research has shown that curcumin reduces tissue peroxidation rates after MI onset while enhancing antioxidant capacity (Rahnavard et al., 2019). Cs exhibits anti-inflammatory, anti-infectious, and anti-tumor effects. In a model of MI induced by LAD in C57BL/6 mice, Cs significantly reduced infarct size compared to the control group. Cs also demonstrated protective effects against hypoxia-induced cardiomyocyte apoptosis by upregulating miR-7a/b expression and downregulating SP1 expression (Geng et al., 2016). In a mouse model of LAD-induced MI, curcumin exhibited a notable decrease in collagen deposition *in vivo*, along with inhibition of proliferation and migration of cardiac fibroblasts and MMP expression. Moreover, curcumin pretreatment attenuated the downregulation of SIRT1 following MI, indicating its potential to prevent myocardial fibrosis induced by MI through SIRT1 downregulation (Xiao et al., 2016). A daily intake of 500 mg of the Curcumin Plus Piperine Supplement for a period of 8 weeks has been demonstrated to improve lipid, liver enzyme and glucose status, as well as significantly reduce myocardial injury in patients with acute MI, according to the findings of clinical studies. However, no effect was observed on ejection fraction and serum troponin I concentration, renal function parameters and electrolytes in patients with AMI (Tabaee et al., 2021). Curcumin nanoparticles (nC) offer superior bioavailability and improve herbs delivery to infarcted myocardial tissues, thereby enhancing antioxidant defenses in cardiomyocytes (Nabofa et al., 2018). Additionally, curcumin plays a vital role in enhancing mitochondrial function in damaged cardiomyocytes (Garvin et al., 2018). A recent study demonstrated that doses of 200 mg/kg of both curcumin and curcumin nanoparticles significantly reduced heart rate before ISO administration and prevented the expansion of QRS complexes after MI. Furthermore, nC was more effective than curcumin in preventing the enlargement of myocardial injury and mitigating interstitial edema and inflammation. In rats with heterozygote-induced MI, nC showed superior efficacy in preventing electrocardiographic and biological changes following MI induction (Boarescu et al., 2019).

#### 6.1.3 Ovothiol-A

The sea urchin is a traditional model organism in developmental biology and is a source of many pharmaceutical products (Sadek et al., 2022). Due to the unique position of the sulfhydryl group on the imidazole ring of histidine, ovothiol (Ovothiol-A), isolated from sea urchins, possesses a distinct antioxidant capacity (Madany et al., 2022). Ovothiol-A exhibits robust antioxidant activity against oxidative stress induced by MI, acting as a dianisidine and protecting cells from reactive oxygen and nitrogen species (Castellano and Seebeck, 2018). Research has shown that administering Ovothiol-A at a dosage of 500 mg/kg for 7 days effectively mitigates MI by exerting antioxidant and anti-inflammatory effects (Mohammed et al., 2022).

#### 6.1.4 Nerolidol

Terpenes, synthesized by plants as secondary metabolites, are among the most studied natural metabolites, playing a pivotal role in cardiovascular disease therapy (Silva et al., 2021). A notable terpene is neuroalool (3,7,11-trimethyl-1,6,10-dodecane-3-ol) (NRD), a sesquiterpene alcohol found in both cis and transforms. Naturally occurring in lavender, lemongrass, and ginger (Chan et al., 2016), NRD exhibits anti-cancer, wound-healing, anti-inflammatory, and antioxidant properties (Baldissera et al., 2018). Administering 100 mg/kg of NRD significantly decreased left ventricular developed pressure (LVDP), reduced malondialdehyde (MDA) levels, increased superoxide dismutase (SOD) activity, and improved cardiac function in rats with MI (Gonçalves et al., 2022). Additionally, short-term NRD administration prevented electrocardiogram changes, indicating a protective effect on cell membrane function (Gonçalves et al., 2022). Another study showed that administering NRD at doses of 100 and 200 mg/kg for 21 days prevented ISO-induced elevation of cardiac and liver marker enzymes (Asaikumar et al., 2019). These findings suggest that sesquiterpenes possess significant antioxidant effects, mitigating myocardial damage caused by ISO and protecting the heart from further harm.

#### 6.1.5 Salvianolate

Salvianolate (Sal), a water-soluble component primarily composed of 80% magnesium B acetate, is derived from the Chinese medicine Danshen and is widely used to treat cardiovascular diseases (Qi et al., 2018). Additionally, Sal is used clinically to address intrastent restenosis caused by vascular remodeling (Zhang M. et al., 2022a). Research by Lin and Zheng (2017) demonstrated that Sal can block apoptosis during MI by downregulating miR-122-5p. Furthermore, Sal reduces the expression of BAX and caspase-3 while enhancing the expression of Bcl-2 through miR-122-5p downregulation, thereby playing an anti-apoptotic role and reducing the MI area (Yue et al., 2017). Sal also prevents myocardial matrix fibrosis by inhibiting heat-related myocardial signaling pathways and downregulating TGF-β1/SMAD2/3 signaling pathways (Yue et al., 2017). Additionally, Sal alleviates MI size, myocardial hypertrophy, and systolic dysfunction through the CAN/NFATc3/BMHC pathway, significantly improving myocardial cell remodeling post-MI (Chen et al., 2022).

#### 6.1.6 β-caryophyllene (BCP)

In recent years, certain G protein-coupled receptors (GPCRs) in the endocannabinoid system, particularly cannabinoid type 1 and type 2 receptors (CB1 and CB2), have emerged as significant therapeutic targets for cardiac protection. CB2 receptors play a pivotal role in treating inflammatory conditions such as atherosclerosis and AMI (Puhl, 2020). Among the ligands interacting with CB2 receptors, BCP has garnered interest due to its widespread presence in essential oils like cinnamon, oregano, black pepper, basil, and cloves. BCP, a sesquiterpene derived from plants and a dietary cannabinoid, exhibits strong full agonist activity on CB2 receptors and holds therapeutic potential for myocardial injury due to its diverse pharmacological properties. Recent findings indicate that BCP alleviates oxidative stress, inflammation, and apoptosis, demonstrating a protective effect against doxorubicin-induced cardiotoxicity (Meeran et al., 2019). Intragastric administration of BCP at a dosage of 50 mg/kg twice daily for 10 days significantly alleviated ISO-induced MI (Meeran et al., 2021b). Additionally, BCP exhibits antioxidant-mediated cardioprotective effects through the modulation of the PI3K/AKT/Nrf2 signaling pathway. The protective role of BCP in rats with ISO-induced MI is dependent on CB2 receptor activity (Meeran et al., 2021b).

#### 6.1.7 Alpha-terpineol (TPN)

Terpineol, an unsaturated monocyclic monoterpene alcohol found naturally in leaves, flowers, fruits, and resin, has attracted significant attention from academia and industry due to its economic importance in pharmaceuticals, agriculture, food, and perfumery. In recent years, global annual sales of terpenoids have surged to approximately $12 billion (Leavell et al., 2016). Consequently, researchers have focused on developing novel cardiovascular herbs derived from terpenes (Alves-Silva et al., 2016). TPN, a volatile monoterpene alcohol and key metabolites of heptapineol resin, has been historically used by Indigenous Brazilian tribes to treat cardiovascular ailments (Marques et al., 2010). Scientific studies indicate that Heptaphyllum C. essential oil, rich in TPN, possesses vasodilatory and hypotensive properties, beneficial for cardiovascular health (Mobin et al., 2017). A recent study found that administering TPN via gavage at doses of 25, 50, and 75 mg/kg for 15 days significantly decreased infarct size in mice with ISO-induced MI. Additionally, mild lactate dehydrogenase leakage indicated TPN’s cardioprotective effects. Furthermore, while ISO increased PRI, animals pretreated with TPN showed a decrease in PRI, enhancing oxygenation of the infarcted heart (Paulino et al., 2022).

#### 6.1.8 Diosmetin

Research indicates that natural products, especially flavonoids, exhibit protective properties against ischemic heart disease (IHD). Several research studies have suggested that flavonoids can effectively reduce the risk of hypertension and CVD. Hesperidin, lignans, and naringenin, among other flavonoids, have been documented to exhibit a range of biological effects, including cardioprotective, antioxidant, and anti-inflammatory properties (Maleki et al., 2019). Diosmetin (5,7,30-trihydroxy-40-methoxyflavone) is a flavonoid present in lemon peel and citrus fruits. Lemon (*Citrus limon* L.), olive (*Olea europaea* L.), and rosemary (*Rosmarinus officinalis* L.) are recognized sources of diosgenin, which has been shown to possess antioxidant and cardioprotective properties. Diosmetin is a crucial phytochemical in these plants, known for its diverse pharmacological effects, including antioxidant, hypolipidemic, anti-inflammatory, antiproliferative, and pro-apoptotic properties (Chen et al., 2020). Previous research has established a connection between these actions and the management of MI. Early studies have suggested the potential cardioprotective benefits of diosmetin (Mo et al., 2020). Moreover, administering 1 and 3 mg/kg doses of diosgenin mitigated ISO-induced increases in ECG t-wave and deep q-wave, along with the extent of MI in rats. Additionally, pretreatment with diosmetin lowered the increase in serum troponin I induced by ISO (Ahmad et al., 2023).

#### 6.1.9 Kaempferol-3-O-rutinoside (KR)

KR can be found in *Carthamus tinctorius* L., *Nymphaea candida*, *Afgekia mahidoliae*, and green tea, showcasing commendable pharmacological properties that offer resilience against hepatic injury, cerebral I/R injury, dementia, hyperglycemia, MI, and Coronary Virus Disease 2019 (Yu et al., 2021). KR confers protection against cerebrovascular injury and mitigates apoptosis induced by cerebral I/R injury through the JAK2/STAT3 pathway (Hu et al., 2017). It was discovered that KR exhibited notable anti-inflammatory effects, substantially enhancing cell survival in lipopolysaccharide-induced inflammatory injury in H9C2 myocardial cells, and markedly decreased the expression levels of key proteins such as TLR4, MyD88, and NF-κB within the TLR4/MyD88/NF-κB signaling pathway (Hua et al., 2021). In a recent study, it was shown that oral administration of KR at a dose of 10 mg/kg daily for 28 consecutive days significantly improved cardiac function, attenuated pathological changes in the heart, reduced excessive collagen deposition in the myocardial interstitium, and inhibited cardiomyocyte apoptosis in rats with AMI. Additionally, KR downregulated the expression of NF-κB p65, NLRP3, ASC, caspase-1 p20, GSDMD, and IL-1β, thereby alleviating ventricular remodeling post-MI through inhibition of the NF-κB/NLRP3/caspase-1 pathway (Hua et al., 2022).

#### 6.1.10 Biochanin-A (BCA)

Numerous scientific studies have emphasized the protective advantages of dietary soy isoflavones in managing hypertension, CVD, and diabetes. These metabolites are renowned for their potent antioxidant properties and are widely acknowledged for their role in preventing metabolic disorders. BCA, a methylated flavonoid metabolite found in red clover, soybeans, alfalfa sprouts, peanuts, and chickpeas, offers various health benefits, such as anticancer, antiallergic, anti-inflammatory, and antihypertensive effects. Furthermore, numerous research studies have illustrated BCA’s substantial antioxidant properties in combating metabolic disorders (Azizi et al., 2014). In a recent investigation, oral administration of BCA at a dose of 10 mg/kg significantly reduced serum levels of marker enzymes (SGOT, SGPT, LDH, CK-MB), as well as cTnI and MDA, showcasing its protective efficacy against ISO-induced MI (Govindasami et al., 2020).

#### 6.1.11 Cycloastragenol

Cycloastragenol is a natural product from the root of *Astragalus membrabaceus* (Fisch.) Bge. Cycloastragenol, a bioactive triterpene aglycone derived from astragaloside IV, has been extensively studied despite its biological function not being fully elucidated. It exhibits a range of biological activities, including anti-inflammatory effects (through inhibition of CD69, CD25, and TXNPT/NLRP3 inflammatory vesicles), (Sun et al., 2017), cardioprotective (through inhibition of AKT1/RPS6KB1) (Wang et al., 2018b), anti-aging, and antifibrotic (Wan et al., 2018). Furthermore, studies have demonstrated that cycloastragenol inhibits the activation of constitutive STAT3 in human gastric cancer cells and enhances paclitaxel-induced apoptosis (Hwang et al., 2019). Recent studies indicate that in rats with LAD-induced MI, administering cycloastragenol via gavage at doses of 5, 10, and 20 mg/kg daily for 35 days significantly ameliorated left ventricular injury caused by MI, attenuated ventricular remodeling, and improved cardiac function in MI-afflicted rats. Additionally, cycloastragenol effectively reduced levels of TNF-α, IFN-γ, and IL-17, and modulated cardiac hypertrophy and remodeling through the RhoA and AKT signaling pathways, offering robust protection against MI (Ren et al., 2020).

#### 6.1.12 S-limonene (SL)

SL, a predominant monoterpene found in essential oils extracted from lemon and orange peels (Bacanlı et al., 2017), has been investigated for its effects. Previous research has demonstrated that D-limonene isomers exhibit anti-apoptotic effects in an ISO-induced MI (Durço et al., 2019). Recent studies have shown that SL, administered orally at a dose of 1 mg/kg daily for 35 days, significantly reduces infarcted and fibrotic areas, and mitigates MI in a rat model induced by LAD occlusion. Moreover, SL enhances GPX and SOD activity, suppresses the increase in Ca^2+^ transient amplitude, restores oxidative balance in the infarcted heart, and alleviates cardiac remodeling post-MI (Rhana et al., 2022). Remarkably, SL reduced ROS production in both cardiac cytoplasm and mitochondria, indicating a significant protective effect against myocardial injury, possibly due to the substantial increase in GPX and SOD enzyme activities (Gordan et al., 2020).

#### 6.1.13 Celastrol

Ranunculin is a powerful bioactive metabolite derived from the root bark of *Tripterygium wilfordii* and *Celastrus orbiculatus*, belonging to the Ranunculaceae family, and exhibits remarkable anti-inflammatory properties (An et al., 2020). Research has shown that celastrol effectively reduces inflammation by inhibiting the K63 deubiquitination of NLRP3 through its interaction with the deubiquitinase BRCC3 (Yan et al., 2021). Moreover, a prior study has documented that celastrol demonstrates anti-inflammatory effects in rats with rheumatoid arthritis by inhibiting the activation of NLRP3 inflammasomes, thereby decreasing the secretion of IL-1β and IL-18 (Jing et al., 2021). Furthermore, celastrol exhibited its capacity to improve cardiac function by suppressing the activation of NLRP3 inflammasomes and decreasing the expression of ASC, cleaved IL-1β, and cleaved IL-18. This effect was corroborated by a notable reduction in post-infarction myocardial fibrosis following administration of celastrol at a dose of 1 mg/kg via oral gavage for 28 days in a rat model of LAD-induced MI (Fan et al., 2023).

#### 6.1.14 Fisetin

Fisetin (3′,4′,7-trihydroxyflavonol) is mainly found in a wide range of vegetables and fruits such as strawberries and onions and has recently emerged as a very promising flavonoid with a variety of beneficial properties (Hu et al., 2020). Extensive research in the past has provided ample evidence regarding the antioxidant and anti-inflammatory properties of fisetin (Zhang et al., 2020). Supplementing with fisetin has been demonstrated to mitigate cardiovascular risk by lowering glucose concentrations and reducing hepatic steatosis via activation of PPAR-γ (Wu et al., 2020). Recent studies have shown that administering fisetin to rat models of MI at a dose of 20 mg/kg via oral gavage for 28 days significantly reduces infarct size, lowers MDA levels, and increases levels of SOD, GSH, and catalase. Additionally, fisetin may activate PPARγ by modulating the NF-κB/MAPK signaling pathway, thereby reducing apoptosis, oxidative stress, and inflammation, ultimately improving tissue damage (Garg et al., 2020).

#### 6.1.15 Indole-3-carbinol (I3C)

I3C, an anticancer metabolite present in cruciferous vegetables, has been reported to be neuroprotective, anti-nephrotoxic (El-Naga and Mahran, 2016), antioxidant and anti-inflammatory (Choi et al., 2018), antiplatelet and antithrombotic (Paliwal et al., 2018). I3C prevents cardiac remodeling and hypertrophy and ameliorates Adriamycin-induced cardiotoxicity by reducing oxidative stress and inflammation (Adwas et al., 2016), and salt-induced cardiac hypertrophy (Akkiraju et al., 2022). Furthermore, I3C improves cardiac function by influencing ECG parameters, hemodynamics, cardiac enzymes (such as LDH, AST, ALT), lipid profiles, and blood glucose levels. It also reduces ISO-induced myocardial levels of MDA, NO, TNF-α, and IL-6, while increasing IL-10 levels. Moreover, it protects myocardial integrity by mitigating platelet aggregation, oxidative stress, inflammation, and apoptosis in rat models (Ramakrishna and Krishnamurthy, 2022).

#### 6.1.16 Nootkatone (NKT)

NKT, a naturally occurring bioactive sesquiterpene abundant in grapefruit, has garnered attention for its notable anti-inflammatory (Kurdi et al., 2018) and anti-apoptotic (Nemmar et al., 2018) properties. Recent studies indicate that administering NKT at a dosage of 10 mg/kg via gavage for 28 days significantly improved hemodynamic parameters in a rat model of ISO-induced MI. Moreover, in this model, NKT mitigates oxidative stress, inflammation, and apoptosis by predominantly modulating alterations in the TLR4/NF-κB/MAPK and PI3K/Nrf2/AKT signaling pathways (Meeran et al., 2021a).

#### 6.1.17 Liensinine (LSN)

LSN, a naturally occurring bisbenzylisoquinoline alkaloid derived from lotus seed embryos, has been demonstrated to possess multiple protective effects against CVD (Chen et al., 2021). Studies have revealed that LSN not only obstructed the adverse activation of Wnt/β-catenin signaling post-MI but also suppressed ROS-induced oxidative damage, highlighting the therapeutic potential of LSN in treating myocardial ischemic injury for the first time (Shen et al., 2023).

#### 6.1.18 Auraptene

Auraptene (7-geranyloxycoumarin) is a prevalent metabolite found in the peel of *Citrus hassaku*, including citrus hasaku, which is a member of the mandarin family and native to Japan. Auraptene has been documented to possess anti-inflammatory effects and neuroprotective properties. Given its long history of consumption as part of citrus fruits, the safety of auraptene has been well-established over millennia (Vakili et al., 2017). Furthermore, it has been observed that auraptene effectively inhibits the inflammatory response induced by adipocytidylic acid by modulating the NF-κB/MAPK signaling pathway (Hsia et al., 2021). Moreover, auraptene, a natural activator of PPAR α, effectively inhibited the hypertrophic response induced by pea protein in cardiomyocytes. A recent study further demonstrated that oral administration of auraptene alleviated myocardial hypertrophy and enhanced contractile function in rats with MI by activating PPAR α-associated genes (Sunagawa et al., 2022).

#### 6.1.19 Tanshinone IIA (T-IIA)

T-IIA, a fat-soluble metabolite found in *Salvia miltiorrhiza*, has been demonstrated to offer benefits in HF owing to its anti-inflammatory and antioxidant properties. It has been employed in the management of CVD (Gao et al., 2012). T-IIA has the capacity to enhance coronary blood flow and ameliorate hypoxia-induced metabolic irregularities in the myocardium, thereby enhancing myocardial resilience to hypoxia. Additionally, T-IIA can diminish the infarct area, enhance myocardial contractility, and stimulate myocardial regeneration (Chen et al., 2015). *In vivo* studies have demonstrated that T-IIA administered orally at 1.5 mg/kg for 28 days significantly enhances EF and FS values, exerting cardioprotective effects via the AMPK/mTOR-mediated autophagy pathway in a rat model of HF induced by LAD ligation (Zhang et al., 2019).

#### 6.1.20 Swertiamarin

Swertiamarin, a naturally occurring plant glycoside found in various plant species of the *Gentianaceae* family, has been studied for its effects on conditions such as hypoglycemia, antioxidant properties, and lipid reduction (Wang et al., 2023). In addition to the effects mentioned earlier, swertiamarin demonstrates a range of pharmacological activities, including antidiabetic, anti-inflammatory, hypolipidemic, hepatoprotective, anti-obesity, anti-malarial, anti-leprosy, antioxidant, and anti-inflammatory properties. Swertiamarin is primarily found in the tropical regions of Asia, South America, and Africa (Muhamad Fadzil et al., 2021). The results showed that administering swertiamarin at doses of 20 and 40 mg/kg for 7 days effectively reduced the levels of TNF-α and IL-6, thereby alleviating MI in rats (Wang et al., 2023). With the continuous development of synergistic agents, natural plant-derived herbs have been receiving a great deal of attention as potential cardioprotective agents (Chang et al., 2020). Swertiamarin is not only involved in preventing tissue damage but also exhibits antioxidant effects, likely attributed to its capacity to mitigate ROS by scavenging them and activating genes responsible for antioxidant enzymes (Wang et al., 2023). Studies have documented that swertiamarin has the capability to activate antioxidant genes under experimental conditions (Wu et al., 2017). Moreover, swertiamarin demonstrates anti-inflammatory effects, which could be attributed to its capacity to induce antioxidant genes, disturb oxidative balance, and prevent the oxidation of cellular macromolecules (Wang et al., 2023).

### 6.2 Medicinal plants targeted for the treatment of MI

In the past 5 years, numerous *in vivo* and *in vitro* experiments have highlighted the anti-MI properties of diverse medicinal plants. This section aims to present the scientific literature’s findings on the anti-MI effects of crude extracts and isolated active metabolites from various medicinal plants (Table 2).

**TABLE 2:** Medicinal plants with therapeutic effects on MI from 2018 to 2023.

No	Medicinal plants	Family	Area or country	Types	Dosages	Models	Pharmacological effect	Medicinal use	References
1	*Syzygium polyanthum*	Myrtaceae family	Distribuito neipaesidel sudest asiatico come Myanmar, Thailandia, Malesia, Singapore Indonesia	*In vivo*	3.6 mg/kg i.g., every day for 14 days	LAD induced MI model	TNF-α and ADAM17 levels ↓	Diabetes, hypertension, gastritis, ulcers, diarrhea, skin diseases, and infections	Hasan et al. (2020)
2	*Kedrostis foetidissima*	Cucurbitaceae	Ethiopia, Sri Lanka, Uganda, Kenya, and western Malaysia	*In vivo*	50 and 100 mg/kg i.g., every day for 45 days	ISO induced MI model	ALT, AST, LDH, and CK activities ↑; cTnT level ↓; TC, TG, PL, FFA, and LDL-C levels ↓; HDL-C level ↑; SOD, CAT and GPX levels ↑; HP and TBARS levels ↓	Common cold, measles, diarrhea, chest pain, and extra articular use in infants with diarrhea	Pavithra et al. (2020)
3	*Rheum turkestanicum*	Polygonaceae	Central Asia and northeast Iran	*In vivo*	100 and 300 mg/kg i.g., every day for 10 days	ISO induced MI model	MDA level ↑; mercaptan level ↓; SOD activity ↑; CK-MB, LDH, and CPK activities ↑	Protective effect of doxorubicin on cardiomyocytes, renal protective effect on gentamicin, reduction of liver mercury toxicity, and anti-diabetic effect	Hosseini et al. (2022)
4	*Alpinia speciosa*	Zingiberaceae	It is native to the East Indies and is widely distributed in South America, Oceania, and Asia	*In vivo*	300 mg/kg i.g., every day for 26 days	ISO induced MI model	CK-NAC and CK-MB activities ↓; infarct size ↓; ventricular wall thickness ↓; heart rate ↓	Antihypertensive, vascular relaxation and diuretic effects	Paulino et al. (2019)
5	*Cucumis sativus L*	Cucurbitaceae	Tropical regions of Africa, Asia, and South America	*In vivo*	50, 100, 150, 200, and 300 mg/kg i.g., every day for 28 days	ISO induced MI model	SBP, DBP, MABP, and BPM ↓; heart weight, heart diameter, left ventricular weight, heart weight index, left ventricular index, tibial length index, and tail length index ↓; CK, CK-MB, cTnT, cTnI, LDH, ANP, and lipid levels ↓; ACE, NO, renin, ALT, AST, IL-6, BNP, and cGMP concentrations ↓; MMP-9 mRNA expression ↓	Hyperlipidemia, diuretics, diabetes, intermittent fever, analgesia, burning and inflammation	Wahid et al. (2022)
6	*Tricyrtis maculate*	Liliaceae	Northwest China	*In vitro*	10, 5, 1 and 0.5 mg/mL incubated for 2 h	—	TNF-α ↓; NO content ↓	Bruising, cough, tuberculosis, and ischemic cerebrovascular disease	Yu et al. (2021)
7	*Artocarpus Altilis*	Artocarpus	Indonesia, Malaysia	*In vivo*	50 and 100 mg/kg i.g., every day for 8 days	ISO induced MI model	MCV, MCH, and MCHC ↑; blood flow ↑; blood viscosity ↑	Cardiovascular efficacies, anticarcinogenic, antimicrobial, antifungal, and anti-inflammatory properties	Thomas et al. (2022)
8	*Sanguisorba. minor*	Rosaceae family	—	*In vivo*	100 and 300 mg/kg i.g., every day for 9 days	ISO induced MI model	MDA ↓; lipid peroxidation and thiol suppression ↓; SOD and CAT ↑; CPK, LDH, CKMB, and cTnT levels ↓; AST and ALT ↓; TG, TC, LDL, and VLDL ↓; HDL ↑; cellular damage ↓	Bleeding, eczema, diarrhea anti-bacterial, neuroprotective, anti-inflammatory, and anti-cancer properties	(Hosseini et al., 2023)
9	*Salvia coccinea leaf*	Lamiaceae family	India	*In vivo*	200, 400 and 600 mg/kg i.g., every day for 14 days	ISO induced MI model	Serum CK, cTnT, cTnI, LDH, AST, and ALT levels ↓; the P wave, QRS complex, and R-R interval ↑; the QT value ↓; heart rate ↓; necrosis, hypertrophy, degeneration, and edematous intermuscular space ↓; SOD, CAT, and GPx antioxidant enzyme levels ↑; MDA level ↓	Diabetes, MI, arthritis, and cataract	Sundaramoorthy and Shanmugam (2023)
10	Ac,ai supplementation	Arecaceae family	A palm fruit native to the Amazon region of Brazil	*In vivo*	2% and 5% ac¸ai pulp feed every day for 90 days	LAD induced MI model	PDH, β-OHADH, CS, and complex I activities ↑; LDH activity ↓; MDA content and SOD activity ↓; IL-10, TIMP-1 concentration, and ICF% ↓	Commonly used in energy drinks, ice cream, fruit juices, pharmaceutical products and cosmetics	Figueiredo et al. (2022)
11	Nutmeg-5	Myristicaceae	Mongolia	*In vivo*	200, 400, and 800 mg/kg i.g., every day for 28 days	LAD induced MI model	HW/BW and HW/TL ↓; infarct size ↓; CK-MB, LDH, and cTnI levels ↓; CAT, GPX, SOD, and GSH activities ↑; mito density ↑; NDUFA5, PINK1, and ACSL1 protein levels ↑; Hif-1α expression ↓	Ischemic heart disease	Liu et al. (2022)
12	Germinated brown rice	—	—	*In vivo*	100 g feeding for 120 days	LAD induced MI model	FS ↑; HR ↓; ventricular arrhythmia, S-T segment shift, and T-wave alternans ↓; infarct size ↓	Anti-atherosclerotic, antidepressant, anti-inflammatory, antidiabetic, and hypolipidemic	Petchdee et al. (2020)
13	Lavender oil	Labiatae	—	*In vivo*	100, 200, and 300 mg/kg i.p.	LAD induced MI model	Infarct size ↓; CK-MB and troponin I levels ↓; GPX and SOD activities ↑; MDA level ↓; TNFα and IL-1β levels ↓; IL-10 levels ↑; DNA fragment and apoptosis activities ↓	Antioxidant, anti-inflammatory and anti-bacterial properties	Souri et al. (2019)
14	Storax	—	—	*In vivo*	0.1, 0.2, and 0.4 g/kg i.g., every day for 7 days	ISO induced MI model	Heart rate, the ST-segment amplitude, and the Q-wave ↓; EF and FS ↑; LVIDs and LVESV ↓; LVDP, LVEDP, -dp/dt max and T ↓; +dp/dt max and V max ↑; AST, LDH, CK-MB, and α-HBDH ↓; myocardial injury and myocardial fibrosis ↓; type 1 and 2 collagen ↓; Bax and cleaved caspase-3 expression ↓; Bcl-2 expression ↑; AT1R, Ankrd1, and P-p53 (ser15) levels ↓; Mdm2 level ↑	CHD, pharmacological effects, such as anti-myocardial ischemia, anti-arrhythmia, anti-platelet aggregation, anti-inflammation, and anti-apoptosis	Xu et al. (2022b)

#### 6.2.1 *Syzygium polyanthum* (SPE)

SPE, commonly known as bay leaf or “*salam*,” is widely distributed in Southeast Asian countries such as Myanmar, Thailand, Malaysia, Singapore, and Indonesia. This ethnomedicinal plant holds significant pharmacological potential for treating various diseases, including diabetes, hypertension, gastritis, ulcers, diarrhea, skin diseases, and infections (Widyawati et al., 2015; Widjajakusuma et al., 2019). Phytochemical screening has revealed that its leaves contain essential oils, tannins, flavonoids, terpenoids, and fatty acids (Widyawati et al., 2015). Administration of SPE via nasogastric tube at a dose of 3.6 mg/kg significantly reduced ADAM17 expression in cardiomyocytes, subsequently affecting TNF-α regulation, and demonstrating SPE’s inhibitory effect on cardiac inflammation post-MI. This suggests that SPE could be a promising future anti-inflammatory agent (Hasan et al., 2020).

#### 6.2.2 Phenolic fraction of *Kedrostis foetidissima* (PFK)

*Kedrostis foetidissima* (Jacq.) Cogn, a cucurbit plant with traditional medicinal significance in Tamil Nadu, is found in Ethiopia, Sri Lanka, Uganda, Kenya, and western Malaysia. Traditionally, it treats ailments like the common cold, measles, and diarrhea (Kamatenesi et al., 2011), and is applied externally on the joints of infants with diarrhea (Ragupathy et al., 2008). Although previous studies have demonstrated its leaves’ *in vitro* antioxidant activity (Kalaisezhiyen et al., 2017), scientific evidence for the cardioprotective effects of its partially purified leaves (PFK) is lacking. Recent findings show that gavage of PFK at doses of 50 and 100 mg/kg for 45 days significantly reduced serum cTnI levels and cardiac biomarker activity in ISO-treated rats, indicating PFK’s protective effect on the myocardium and its ability to prevent myocardial injury (Pavithra et al., 2020). PFK also enhances antioxidant effects by increasing enzyme levels and activities, significantly restoring CAT, GPX, and SOD levels, and preventing myocardial damage by scavenging free radicals (Pavithra et al., 2020).

#### 6.2.3 Rheum turkestanicum

*Rhubarb*, a member of the Polygonaceae family, is a commonly used spice in TCM. Its active metabolite include anthraquinones, glycosides, and tannins (Ghorbani et al., 2019). Anthraquinone derivatives such as *rhubarbine*, rhodopsin, rhubarb phenol, chymosin, citrinin, and aloe rhubarbin are prominent in the genus *Rhubarb* (Ghorbani et al., 2019). *Rheum turkestanicum* Janisch, or Turkish rhubarb, primarily grows in central Asia and northeastern Iran (Ghorbani et al., 2019). The extract contains anthraquinones (such as hordenine derivatives), fatty acids (including 9-octadecenoic acid), and flavonoids (like epicatechin and quercetin). In a rat model of MI, administering 100 and 300 mg/kg of *R. turkestanicum* via gavage prevented MI by reducing oxidative stress and lipid peroxidation, increasing antioxidant enzyme levels, and lowering serum LDH, CPK, and CK-MB levels (Hosseini et al., 2022).

#### 6.2.4 *Alpinia speciosa* (AZE)

*Alpinia zerumbet* (Pers.) B.L. Burtt. & R.M. Smith, also known as *Alpinia speciosa*, is a member of the ginger family indigenous to the East Indies and widely found in South America, Oceania, and Asia. In Brazil, it is commonly referred to as “colônia” and is widely used in traditional medicine for its hypotensive and diuretic properties, often in the preparation of wine and tea using its leaves and flowers (Cartaxo et al., 2010). Several cardiovascular effects, such as antihypertensive, vasorelaxant, antioxidant properties, antiplatelet activity, and cardioinhibitory effects, have been reported for *A. zerumbet* extract. Continuous gavage of AZE at a dose of 300 mg/kg for 26 days significantly reduced serum CK-NAC and CK-MB activities in ISO-treated rats, indicating its cardioprotective effects (Paulino et al., 2019). The cardioprotective mechanism of AZE may involve the cardiac inhibitory effect caused by L-type Ca^2+^ current blockade and its antioxidant properties. Additionally, long-term treatment with the extract prevented ISO-induced cardiac hypertrophy (Paulino et al., 2019).

#### 6.2.5 *Cucumis sativus* L. (CSL)

CSL, a member of the *Cucurbitaceae* family, is a widely cultivated edible vegetable, particularly in tropical regions of Africa, Asia, and South America. It is often served as a green appetizer and incorporated into regular meals due to its rich content of vitamins, proteins, carbohydrates, and fatty acids (Barua et al., 2014). In Pakistan and India, the seeds of CSL are frequently used to manage gastrointestinal and urinary concerns, as well as to enhance heart function. Furthermore, the fruits are applied to alleviate burning sensations, burns, and open ulcers. Research has unveiled a spectrum of beneficial properties associated with CSL, encompassing antioxidant, anti-ulcer, cytotoxic, anti-inflammatory, antihypertensive, and analgesic effects. The seeds of CSL chinensis are utilized for managing conditions such as hyperlipidemia, promoting diuresis, addressing diabetes mellitus, and intermittent fever, providing analgesia, alleviating burning sensations, and reducing inflammation (Wahid et al., 2022). CSL seeds and fruits contain volatile oil, fixed oil, flavonoids, steroids, flavonoids, tannins, and phytosterols (Wahid et al., 2021). Research suggests that CSL.EtOH may exert potent antihypertensive effects in normotensive rats and provide protection against MI by modulating endothelium-derived relaxing factors and reversing changes in amino acid metabolism, energy metabolism, and oxidative stress associated with MI (Wahid et al., 2022).

#### 6.2.6 *Tricyrtis maculate* (TSM)

Lily of the valley *Tricyrtis maculata (D. Don)* Machride, is primarily distributed in northwest China and is a member of the lily family. It is commonly employed in treating bruises, coughs, tuberculosis, and ischemic CVD. Studies have demonstrated that extracts of TSM notably enhance microcirculation, prevent thrombosis, and alleviate blood stasis (Vakili et al., 2017). Nicotiflorin, a flavonoid glycoside derived from TSM, exhibits a range of pharmacological benefits such as anti-inflammatory, antioxidant, antibacterial, antiviral, analgesic, and neuroprotective properties (Wang L. et al., 2021a). Research has shown that TSM combats AMI by modulating the AKT/FoxO/Bcl signaling pathway, improving circulation balance, reducing inflammatory responses, preventing cardiomyocyte apoptosis, and regulating energy metabolism (Yu et al., 2021).

#### 6.2.7 *Artocarpus Altilis* (AA)

AA contains abundant vitamins, essential minerals, and medicinal plant known for their anticancer, antibacterial, antifungal, and anti-inflammatory properties, as well as their ability to support cardiovascular health (Cifuentes et al., 2016). Studies have revealed that AA enhances endothelial cell function by antagonizing alpha-adrenergic receptors and Ca^2+^ channels, resulting in a negative chronotropic effect (Nwokocha et al., 2020). Recent studies have demonstrated that administering AA via gavage at doses of 50 and 100 mg/kg improved blood flow reduction and mitigated MI in a rat model induced by ISO. Additionally, AA enhances oxygen availability, lowers blood viscosity, and promotes blood flow by influencing endothelial function and NO availability (Thomas et al., 2022).

#### 6.2.8 *Sanguisorba. minor* (*S. minor*)

*Sanguisorba minor*, a member of the *Rosaceae* family, is a plant known for its diverse biological activities, which include antioxidant properties. It is frequently utilized in the treatment of conditions such as bleeding, eczema, diarrhea, and various other ailments (Zhao et al., 2017). Various phytochemicals known for their ability to counteract oxidative stress, including phenols, flavonoids, and terpenoids, have been isolated from the Sanguisorba genus (Zhao et al., 2017). Specifically, *S. minor* demonstrates a broad spectrum of pharmacological activities including antioxidant, antimicrobial, neuroprotective, anti-inflammatory, and anticancer properties. Its antioxidant effects are mainly attributed to its abundant polyphenol content (Cirovic et al., 2020). Recent research has shown that administering an aqueous ethanolic extract of *S. minor* orally at doses of 100 or 300 mg/kg for nine consecutive days elevated levels of SOD and catalase, reduced MDA levels, and decreased cardiac markers such as cTnT, LDH, CK-MB, and CPK. This effectively mitigated myocardial ischemia induced by ISO (Hosseini et al., 2023).

#### 6.2.9 Aqueous extract of *Salvia coccinea* leaf (AESL)

*Salvia* L., belonging to the family *Labiatae*, encompasses annual, biennial, or perennial herbs as well as woody subshrubs and represents the largest genus of plants within this family. Numerous species and varieties of *Salvia* have been utilized in traditional medicine. Several studies have extensively explored the antioxidant properties of *S. palaestina* and *S. ceratophylla*, while *Salvia officinalis*, native to the Middle East and Mediterranean region, has been confirmed for its antidiabetic effects in rat models (Brito-da-Costa et al., 2021). Furthermore, investigations into the cardioprotective effects of *S. syriacaof* from Turkey, *S. miltiorrhiza* from China, and the therapeutic potential of *Salvia divinorum* for vascular diseases such as atherosclerosis have also been conducted (Brito-da-Costa et al., 2021). These findings collectively demonstrate the widespread utilization of Salvia species in biological research (Khattab and Mohamed, 2012). Moreover, AESL has shown significant promise in alleviating diabetes in rats, positioning it as a potential phytopharmaceutical source for various inflammatory diseases and as a potent antioxidant and free radical scavenger (Sudaramoorthy et al., 2021). Recent studies have revealed that administering 200, 400, and 600 mg/kg of AESL via gavage significantly reduced the risk of ISO-induced MI and mitigated the elevation of blood pressure in a rat model of ISO-induced MI. Additionally, AESL demonstrated the ability to scavenge free radicals and ROS, thereby inhibiting the expression of various inflammatory molecules and attenuating MI by interfering with NF-κB nuclear translocation (Sundaramoorthy and Shanmugam, 2023).

#### 6.2.10 Ac¸ai supplementation (ACS)

Ac¸ ai (*Euterpe oleracea Mart*.) is a palm plant indigenous to the Amazon region of Brazil, bearing palm fruits. The seeds of ac,ai find utility in animal feed, agricultural plantations, and domestic gardens, while the pulp of ac,ai serves as a functional food metabolite incorporated into energy drinks, ice creams, juices, pharmaceuticals, and cosmetics (de Moura and Resende Â, 2016). Notably, the global consumption of ac,ai pulp has been steadily increasing. Furthermore, hydroalcoholic seed extract has been utilized in experiments as a supplement derived from ac,ai seeds (Zhou J. et al., 2018a). Given that ac,ai seeds are not meant for consumption, ac,ai capsules have been employed for supplementation in clinical trials. Moreover, ac,ai pulp, being edible and appealing in appearance, texture, and flavor, is a convenient option for use in both clinical and experimental studies compared to seeds (Fragoso et al., 2018). In a particular study, the supplementation of 5% ac,ai pulp over six consecutive weeks resulted in a significant enhancement of energy metabolism and a reduction in oxidative stress (Alegre et al., 2020). During normal physiological conditions, fatty acids serve as the primary energy source. Following an MI, calcium supplementation enhances fatty acid oxidation while reducing glycolytic pathways. Ac,ai may mitigate cardiac remodeling post-MI in rats by alleviating oxidative stress, enhancing energy metabolism, modulating inflammation, and decreasing fibrosis (Figueiredo et al., 2022). Additionally, ACS reduced malondialdehyde MDA concentration in the heart, thereby ameliorating oxidative stress. In addition, ACS reduces SOD activity, which reduces extracellular matrix degradation and thus reduces fibrosis (Figueiredo et al., 2022).

#### 6.2.11 Nutmeg-5 (Nu5)

Nutmeg, an ancient aromatic spice, holds a rich history of utilization in traditional medicinal practices across regions such as Thailand, Myanmar, Malaysia, Vietnam, and India (Barman et al., 2021). The seeds of Nutmeg (Myristica fragrans) fruits, referred to as nutmeg in Chinese medicine, exhibit a plethora of pharmacological properties including anti-inflammatory, analgesic, antioxidant, anti-obesity, anti-diabetic, and cardioprotective effects. These beneficial attributes are attributed to a diverse range of phytochemicals present in nutmeg, such as lignans, neo-lignans, dibenzo-phenanthroids, phenylpropanones, terpenoids, alkanes, fatty acids, and fatty acid esters (Ha et al., 2020). Nu5 represents a time-honored and traditional formula within Mongolian medicine, widely employed for the management of IHD. This formulation, listed in the Drug Standard of the Ministry of Health of the People’s Republic of China (Mongolian Medicine), comprises nutmeg (*Myristica fragrans* Houtt. seed), *Aklandia lappa* Decne. root, *Inula helenium* L. root, *Fructus Choerospondiatis* seed, and *Piper longum* L. fruit. Prior web-based pharmacological assessments have highlighted the protective effects of nutmeg and Myristica fragrans on cardiac tissue against isoprenaline-induced MI in rats by mitigating cardiomyocyte apoptosis, oxidative stress, and inflammatory responses (Lu et al., 2018). Moreover, Nu5 has demonstrated the ability to suppress cardiac HIF-1α expression, enhance cardiac metabolism, address mitochondrial dysfunction, and thereby alleviate cardiac remodeling in mice post-MI. Furthermore, the presence of numerous antioxidant-rich flavonoids in Nu5 has been shown to attenuate oxidative stress induced by LDA ligation (Liu et al., 2022).

#### 6.2.12 Germinated brown rice (GBR)

In recent times, there has been a rising trend in the consumption of diets and dietary metabolites known for their beneficial effects (Morabbi Najafabad and Jamei, 2014). Specifically, the capacity of whole grain foods, particularly GBR, to confer protective benefits against metabolic diseases has garnered attention in the realm of whole grain food consumption (Lillioja et al., 2013). The process of germination is a natural phase in the growth of seeds, contributing to an improved texture and the addition of valuable metabolites to the seed (Imam et al., 2013). GBR represents a biotransformed product derived from brown rice and is recognized for its remarkable health-enhancing and disease-preventing properties (Ho et al., 2012). Numerous studies have underscored the diverse pharmacological activities associated with GBR, including its anti-atherosclerotic, antidepressant, anti-inflammatory, antidiabetic, and hypolipidemic properties, thereby positioning it as a rich source of phytochemicals (Shen et al., 2016). It was found that GBR intake significantly reduced heart rate and improved cardiac functions such as LVFS in a rabbit model of chronic MI and was cardioprotective in a rabbit model of chronic MI (Petchdee et al., 2020).

#### 6.2.13 Lavender oil (LO)

Lavender, a member of the *Labiatae* family, is valued not only for its therapeutic characteristics but also for its various cosmetic applications (Vakili et al., 2014). LO, extracted from the flowers of Lavandula angustifolia, contains numerous active metabolite, including linalyl acetate, linalool, 1,8-cineole, lavandinol, lavandula angustifolia acetate, camphor, cis-beta-ocimene, trans-beta-ocimene, 1-terpinen-4-ol, α-pineneol, limonene, tannins, coumarins, flavonoids, phytosterols, and triterpenoids (Verma et al., 2010). LO exhibits potent antioxidant, anti-inflammatory, and antibacterial properties (Hajiali et al., 2016). Furthermore, research indicates that the antioxidant metabolites present in LO may possess anti-inflammatory activity (Silva et al., 2015). In a rat model of LAD-induced MI, gavage of 100, 200, and 300 mg/kg of LO effectively reduced myocardial infarct size, as well as CK-MB and troponin I levels. In addition, LO significantly inhibited apoptosis by down-regulating pro-inflammatory cytokines and up-regulating anti-inflammatory cytokines and restored endogenous antioxidant defenses after MI (Souri et al., 2019).

#### 6.2.14 Storax

Storax is an aromatic resin derived from the trunk of *Liquidambar orientalis* Mill. and processed into a semi-fluid concentrate as per the National Pharmacopoeia Committee (2020), belongs to the witch *hazel* family. Its well-established efficacy in treating coronary heart disease has led to widespread use and documentation in the medical practices of numerous countries, including its inclusion in the U.S. Pharmacopoeia (2017). A multitude of clinical formulations, such as Suhexiang pills and Guanxin Suhe pills, containing astragalus hubcaps strong heart pills, are extensively employed for the treatment of coronary artery disease, delivering notable efficacy, minimal adverse effects, and high patient compliance, thus presenting promising application prospects (Cheng et al., 2023). Furthermore, contemporary pharmacological investigations have indicated the broad applicability of storax in managing cardiovascular ailments, demonstrating pharmacological effects encompassing anti-myocardial ischemia, anti-arrhythmia, anti-platelet aggregation, anti-inflammatory, and anti-apoptosis properties (Mu et al., 2023). Recent research has revealed that in an ISO-induced MI model, oral administration of storax at 0.2 g/kg significantly reduced heart rate and effectively mitigated ISO-induced myocardial injury and myocardial fibrosis in rats. Moreover, storax exhibited the ability to ameliorate myocardial diastolic dysfunction, preserve myocardial morphology and pumping function, notably decrease the deposition of collagen types 1 and 3 in AMI rats, and alleviate cardiomyocyte apoptosis in ISO-induced post-infarction myocardial fibrosis (Xu Z. et al., 2022b).

## 7 Clinical evaluation

Although many natural products have been used to treat and improve MI as well as its complications, randomized controlled trials are limited. In a clinical trial testing a freeze-dried lingonberry dietary supplement to improve walking distance and lipids after MI, a 40 g/d dose of Freeze-dried bilberry (Vaccinium myrtillus) was administered to 50 MI exchange patients, who, after 8 weeks of intervention, showed a significant increase in the mean 6-min walking test distance and a significant decrease in isolated oxidized low-density lipoproteins *in vivo*, demonstrating that Freeze-dried bilberries may have a clinically relevant and beneficial effect after AMI (Arevström et al., 2019). Among 60 NSTE-ACS patients treated with elective PCI, 53 (88.3%) had no or mild myocardial injury, and 7 (11.7%) had a severe myocardial injury or MI after clinical use of tanshinolates. The results suggest that tanshinolate treatment can reduce PCI-related severe myocardial injury or MI and open a new way to improve PCI-related severe myocardial injury or MI (Ou et al., 2020). In a randomized controlled trial involving obese women diagnosed with FA, the supplementation of BCP showed potential benefits in enhancing YFAS-S. Specifically, after 8 weeks of BCP administration, participants exhibited improved β-stigmasterol levels compared to those receiving a placebo (Alizadeh et al., 2022). Furthermore, the combination of BCA with Acetyl tetrapeptide-3 and Ginseng Extracts could be a promising phytomedicine for treating androgenetic alopecia. This could be supported by a 24 weeks, triple-blind, randomized controlled research trial (Lueangarun and Panchaprateep, 2020). Several natural products are showing excellent efficacy in treating patients with MI and its complications. They are emerging as promising clinical choices for anti-MI herbs. Tanshinone IIA sodium sulfonate has been demonstrated to have favorable clinical efficacy in the treatment of coronary artery disease. This has the potential to significantly improve patients’ heart rate variability and reduce NT-proBNP levels (Wang and Pan, 2020). A daily intake of 500 mg of the Curcumin Plus Piperine Supplement for a period of 8 weeks has been demonstrated to improve lipid, liver enzyme and glucose status, as well as significantly reduce myocardial injury in patients with AMI, according to the findings of clinical studies. However, no effect was observed on ejection fraction and serum troponin I concentration, renal function parameters and electrolytes in patients with AMI (Tabaee et al., 2021).

## 8 Toxicity and side effects

The toxicological problems associated with herbal medicines are a major public health issue. It is therefore necessary to elaborate on the safety of natural products. Traditionally, the use of clinical doses of metabolites and medicinal plants for the treatment of MI has been considered quite safe. Nonetheless, only a limited number of studies have thoroughly and precisely evaluated the toxicity of natural products, as well as their crude extracts or active metabolites. Hence, it is crucial to exercise significant caution when employing substantial doses of these natural products in traditional medicine, as they may lead to side effects or other adverse reactions. In a study, AE was administered to zebrafish at a concentration of 0.01505 mg/mL for 72 h. The findings suggested that AE might induce hepatotoxicity by activating the NF-κB inflammatory pathway and the P53 apoptotic pathway in zebrafish, confirming its potential hepatotoxic effect (Quan et al., 2019). In acute toxicity studies in mice and Beagle dogs using Salvianolic acid A at doses of 80 or 300 mg/kg, focal necrosis of hepatic and renal tubular epithelial cells, decrease in the relative weight of the thymus gland, and abnormal changes in biochemical parameters were observed 28 days after administration. It suggests that attention should be paid to the examination of liver and kidney functions when Salvianolic acid A is applied clinically (Yang et al., 2022). Exposure to BCP caused genotoxic and cytotoxic damage that affected the growth and survival of nightshade moth larvae (Lepidoptera noctuosa) when fed at various concentrations (5, 25, 125, 625, and 3,125 ppm). This suggests that BCP has some genotoxic and cytotoxic properties, but it can also be an effective alternative to conventional and synthetic insecticides to some extent (Mahajan et al., 2022). Indeed, there have been limited studies examining the side effects of therapeutic doses, with a predominant focus on acute toxicity. For instance, in a trial investigating the developmental toxicity of Swertiamarin in zebrafish, it was observed that Swertiamarin exhibited safety only at lower concentrations (40 µM).

It is worth noting that even herbal can pose a risk of toxicity to humans when administered at higher doses. Hence, it is essential to evaluate the toxicity of both synthetic and natural herbs prior to their human usage (Perumal et al., 2021). In the acute toxicity test, mice received *R. turkestanicum* root extract orally at a dose of 300 mg/kg for 14 days, with no mortality or signs of toxicity observed. In the subacute toxicity evaluation, rats receiving *R. turkestanicum* root extract at 400 mg/kg via gavage for 28 days exhibited no notable alterations in body weight, general behavior, hematological parameters, serum biochemical factors, or histopathology of the heart, liver, kidney, and brain. To sum up, the experiments indicate that short-term administration of *R. turkestanicum* at 400 mg/kg does not seem to induce significant toxicity (Jahani Yazdi et al., 2020). Although natural products, medicinal plants, and their metabolites are known for their robust protective effects against MI and its complications, there is a paucity of research on their toxicity. Consequently, it is imperative to conduct quantitative assessments in future studies to thoroughly investigate the toxicity of these natural products and medicinal plants.

## 9 Conclusions and perspectives

MI is a prevalent global disease with high mortality rates. Its occurrence and progression involve complex and diverse mechanisms, necessitating tailored treatments. For acute ST-segment elevation, prompt reconstruction of the infarcted artery through percutaneous coronary intervention (PCI) is recommended. In contrast, coronary artery bypass grafting (CABG) benefits patients with reduced left ventricular ejection fraction (Neumann et al., 2019; Navarese et al., 2021). However, postoperative complications and rejection can lead to poor prognoses, with significant complications documented in approximately 20% of patients following cardiac surgery (Milojevic et al., 2021). Additionally, conventional herbs such as antiplatelet agents and statins face limitations due to non-targeted distribution, short half-life, and side effects (Han et al., 2018; Luan et al., 2021; Lv et al., 2022). MI mechanisms include oxidative stress, inflammation, apoptosis, and autophagy, and its incidence has been rising over the years. Despite this, no specific herbs currently exists to enhance cardiac function and alleviate ventricular remodeling post-MI. Thus, developing novel therapeutic agents for MI treatment is essential.

Given the bottleneck in chemical synthetic herbs research, the focus has shifted to developing herbs based on natural products (Qian et al., 2024; Xie et al., 2024). Natural products offer a new approach to safer and more effective MI management and treatment. These natural metabolites can target various pathogenic factors through multiple pathways and systems. For example, AS-IV inhibits oxidative stress, reduces inflammation, regulates energy metabolism, and restores cardiac balance (Zhang X. et al., 2022b). Similarly, Cs improves left ventricular systolic function, reduces heart weight, and mitigates inflammation, oxidative stress, and apoptosis (Boarescu et al., 2019). These findings highlight the feasibility and advantages of using natural products to improve MI complications and comorbidities. However, further improvements are needed to facilitate the discovery of new natural medicine therapies.

Firstly, although many natural products and medicinal plants have been shown to have great therapeutic potential in the treatment of MI, comprehensive research on their phytochemical and pharmacological characteristics remains scarce. Future studies should focus on identifying and isolating metabolites with therapeutic activity against MI and elucidating their specific mechanisms of action while excluding subjective evaluation unless indicated.

Secondly, Several Chinese medicine monomer metabolites have shown efficacy in preventing MI, yet factors such as low oral bioavailability restrict their utilization. For instance, curcumin exhibits low oral bioavailability. To advance the development of new metabolites for MI treatment, it is necessary to chemically modify active monomeric metabolites, such as NRD, Sal, BCP, and TPN, to clarify the conformational relationships of their derivatives. Investigating the molecular mechanisms of these phytopharmaceuticals in MI and its complications from a chemical perspective is crucial for early clinical application.

Thirdly, to further the advancement of new metabolites for treating MI, potential remedies such as NRD, Sal, BCP, and TPN have been chemically modified to clarify the conformational relationships of their derivatives. However, global research on the clinical efficacy of medicinal plants in managing MI remains limited. International studies have shown that natural herbs and medicinal plants achieve good results in inhibiting MI by targeting molecular mechanisms such as inflammatory response, oxidative stress, apoptosis, myocardial fibrosis, and angiogenesis regulation.

As the popularity of natural herbs increases and the efficacy of conventional chemical drugs is increasingly questioned, it seems likely that natural herbs will become a more widely used treatment option in the future. It is important to acknowledge that the current body of research is largely confined to cell cultures and animal models. It is essential to consider the safety and tolerability of these substances, as well as their clinical efficacy, to develop more promising treatments. It is therefore evident that further clinical studies are required in order to elucidate the mechanisms and safety of herbal medicines, as well as to develop superior drugs and treatments with the aim of improving human life. Concurrently, it is hoped that the implementation of additional phytochemical screening, biological studies, and the utilization of advanced technologies and innovative approaches will facilitate the unbiased evaluation and review of these herbs and metabolites. It is also noteworthy that the discovery of bioactive metabolites derived from natural products and the exploration of their functions and mechanisms represents a challenging yet highly intriguing task.

## Abbreviations

AA, Artocarpus Altilis; ACS, Ac,ai supplementation; AKT, protein kinase B; AESL, Salvia coccinea leaf; AMPK, adenosine 5′-monophosphate-activated protein kinase; Ang II, angiotensin II; ATP, adenosine triphosphate; AZE, Alpinia speciosa; BCA, Biochanin-A; BCP, β-caryophyllene; CSL, Cucumis sativus L.; Gal-3, Galactaglutinin-3; GBR, Germinated brown rice; I3C, Indole-3-carbinol; IGF-I, Insulin-like growth factor I; IHD, ischemic heart disease; ISO, Isoprenaline; KR, Kaempferol-3-O-rutinoside; LO, Lavender oil; MI, myocardial infarction; MMPs, matrix metalloproteinases; mTOR1, mammalian target of rapamycin 1; NKT, Nootkatone; NRD, Nerolidol; Nu5, Nutmeg-5; PFK, Phenolic fraction of Kedrostis foetidissima; ROS, reactive oxygen species; *S. minor*, Sanguisorba. Minor; SL, S-limonene; SPE, Syzygium polyanthum; T-IIA, Tanshinone IIA; TCM, traditional Chinese medicine; TPN, Alpha-terpineol; TSM, Tricyrtis maculate.
