# Supplementary material for: Research progress on the natural products in the intervention of myocardial infarction
Source: Front Pharmacol. 2024 Aug 22;15:1445349. doi: 10.3389/fphar.2024.1445349 (PMC11374734; doi:10.3389/fphar.2024.1445349)
Supplement: Supplementary file 1 [file DataSheet2.PDF]

ConPhyMP checklist of items for conducting and reporting analytical methods[1,2] relevant for extract type B (for species or botanical drugs widely used or traded without a monograph in a national or international pharmacopoeia)

SECTION/TOPIC	ITEM NO.	CHECKLIST ITEM	YES	NO	NOT APPLICABLE	PAGE NO., IF ANY
Type of extract	1	B – Confirm that the species or botanical drug under investigation is a widely used or traded one without a monograph in a national or international pharmacopoeia.	✓			
Preferred/ main methods for extract characterisation/ chemical analysis	2	(a) Triple chemical fingerprinting methods, each with one or more detection parameters.			✓	
		(b) Quantification of at least two marker compounds (unless this is not feasible, evidence needs to be provided), and justification of the choice of markers (if applicable).			✓	
Alternative methods for extract characterisation/chemical analysis	3	(a) Single chemical fingerprinting method, three different detection parameters should be provided (i.e., altered detection parameters, like TLC/HPTLC with different staining reagents and/or UV excitation wavelengths, HPLC-DAD/LC-DAD with different wavelengths). The same applies to coupling MS or NMR to chromatographic techniques.			✓	
		(b) Quantification of at least two marker compounds (unless this is not feasible, evidence needs to be provided), and justification of the choice of markers (if applicable).			✓	
Use of reference standards	4	(a) Direct overlay of the chromatogram of the sample with that of an officially specified reference standard (if applicable)			✓	
		(b) Chromatographic fingerprinting: Direct overlay of the chromatogram of the sample with that of official reference standards of the powdered plant material or the dry extract from the plant material.				
Comparison of different extracts/ samples of the same plants	5	(a) Direct comparison of the chromatographic/spectroscopic system and/or scoring system for "similarity" to be followed.			✓	

Note: Please also include here the following information about your submitted manuscript:

Name of the journal: Frontiers in Pharmacology

Date of the enquiry: 2024.7.15

Title of the manuscript: Research progress on the natural products in the interventic

List of the authors: Qiuting Guo, Jinhui Wang, Caixia Ni, Jiaojiao Pan, Junbo Zou

[1] Please acknowledge/cite this as follows: Heinrich M, Jalil B, Abdel-Tawab M, Echeverria J, Kulić Ž, McGaw LJ, et al. Best Practice in the chemical characterisation of extracts used in pharmacological and toxicological research—The ConPhyMP—Guidelines. Frontiers in Pharmacology. 2022;13:953205. https://doi.org/10.3389/fphar.2022.953205

[2] We strongly recommend reading this checklist in conjuncton with ConPhyMP 2022 explanaton and elaboraton for important clarifications on all items. If relevant, we also recommend after reading Heinrich et al. (2020) Best practice in research—Overcoming common challenges in phytopharmacological research. Journal of Ethnopharmacology. 2020;246:112230. https://doi.org/10.1016/j.jep.2019.112230
